# Catalytic Hairpin Assembly‐Propelled Weak‐Inputs‐Strong‐Outputs (CP‐WISO) DNA Logic Nanodevices with Orthogonal Design and Contrary Logic Responses

**DOI:** 10.1002/advs.202501430

**Published:** 2025-06-09

**Authors:** Xujuan Lv, Jiawen Han, Juan Wang, Zhihua Lv, Baojian Huang, Shuai Qin, Xuxin Yan, Shaojun Dong, Daoqing Fan

**Affiliations:** ^1^ Key Laboratory of Marine Drugs Ministry of Education School of Medicine and Pharmacy Ocean University of China Qingdao Shandong 266003 China; ^2^ Laboratory for Marine Drugs and Bioproducts Qingdao Marine Science and Technology Center Qingdao Shandong 266237 China; ^3^ Intelligent Wearable Engineering Research Center of Qingdao Research Center for Intelligent and Wearable Technology College of Textiles and Clothing State Key Laboratory of Bio‐Fibers and Eco‐Textiles Qingdao University Qingdao 266071 China; ^4^ State Key Laboratory of Electroanalytical Chemistry Changchun Institute of Applied Chemistry Chinese Academy of Sciences Changchun 130022 China

**Keywords:** catalytic hairpin assembly, contrary logic responses, CP‐WISO DNA logic nanodevices, even‐odd parity generator/checker, molecular diagnostics

## Abstract

Developing innovative multi‐channel DNA logic systems with strong responses for weak inputs is of paramount significance for versatile molecular computing, information processing, smart biosensing, and early medical diagnostics. However, most previous works usually necessitate abundant inputs equivalent to signal probes or computing elements, and present only single‐channel outputs, causing DNAs’ over‐consumption, limited computing functions, flexibility, and maneuverability. Herein, assisted with a catalytic‐hairpin‐assembly (CHA) amplifier and rational orthogonal design, the first universal weak‐inputs‐strong‐outputs DNA system (named CP‐WISO) with contrary logic responses is reported, which exhibited noticeable advantages over previous works. 1) Excellent FRET effect of dual‐signal DNA probes (Cy3/Cy5) brought enriched contrary logic output channels; 2) One‐step CHA amplification avoided DNAs’ over‐consumption, high cost, and complexity; 3) A library of CP‐WISO DNA logic nanodevices with the manipulated orthogonal design is realized, accompanied with enhanced flexibility, maneuverability, and computing proficiency; 4) Even and odd parity generators/checkers (pG/pC) are concurrently operated based on the “XOR∧XNOR”‐type CP‐WISO nanodevice, ensuring the recognition of erroneous bits and secure molecular data transmission; 5) Additionally, taking miRNA‐499, miRNA‐21 and miRNA‐122 as alternative model targets, the “YES∧NOT” and “AND∧NAND” CP‐WISO nanodevices achieved intelligent identification and ratiometric fluorescent detection of miRNAs in human serums, promoting the early diagnostics of acute myocardial infarction and liver cancer.

## Graphical abstract

Catalytic hairpin assembly‐propelled weak‐inputs‐strong‐outputs (CP‐WISO) DNA logic nanodevices with orthogonal design and contrary logic responses are innovatively fabricated. Moreover, the even and odd parity generators/checkers for ensuring normal molecular data transmission, and the logic‐programmed miRNA biosensors with ratiometric fluorescent responses are further realized based on the universal CP‐WISO platform, promoting the early diagnostics of major diseases.

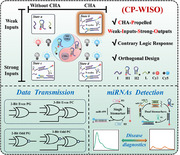

## 1 Introduction

Facing the contradiction between the gradually approached integration/miniaturization limits of semiconductor transistors and people's growing demand, biocomputing was explored and taken as a powerful substitute for silicon counterparts. Since Prof. de Silva created the first molecular AND logic gate in 1993,^[^ ^1^ ^]^ more and more biocomponents, such as enzymes, peptides, proteins, and nucleic acids, have been introduced to the area of molecular computing.^[^ ^2^, ^3^ ^]^ Representatively, DNA, as the carrier and transmitter of genetic information, has evoked revolutionary advancements with the help of burgeoning DNA nanotechnology.^[^ ^4^, ^5^, ^6^, ^7^, ^8^ ^]^ In recent decades, varieties of DNA logic devices (such as encoder/decoder, adder/subtractor, keypad lock, parity generator/checker (pG/pC), voter, multi‐valued logic) with emerging computing functions that could present optical/electrochemical outputs have been designed.^[^ ^9^, ^10^, ^11^, ^12^ ^]^ Additionally, stimuli‐responsive DNA logic nanodevices have also been delicately designed and applied to subcellular imaging, drug delivery, CRISPR‐Cas genome editing, molecular diagnostics, and programmed nano‐catalytic therapy.^[^ ^13^, ^14^, ^15^, ^16^, ^17^, ^18^, ^19^ ^]^ Whereas, we cannot ignore the serious challenges in this area while facing with the gratifying progress.

Notably, previous DNA logic systems usually require the trigger of abundant inputs equivalent to signal probes or computing elements, resulting in the over‐consumption of DNAs, low computing efficiency, limited flexibility, and maneuverability. Aiming at surmounting this problem, Huang's group proposed the concept of “weak‐inputs‐strong‐outputs” (WISO).^[^ ^20^ ^]^ By integrating toehold‐mediated‐strand‐displacement with a hybridization chain reaction, they established several logic gates and number classifiers using low concentrations of inputs. However, there still exist non‐negligible drawbacks that remain to be conquered. First, the individual WISO logic gates with different functions require reoperation, and the problem of DNAs’ high consumption was not evidently solved. Second, all the WISO logic gates presented only single‐channel output, inducing restricted computing functions and the deficiency of hierarchical control. Last but not least, the excellent sensitivity toward weak inputs and digital outputs of WISO DNA nanodevices could work as an ideal toolbox for accurate logical diagnostic scenarios, yet barely reported before. Therefore, exploring novel strategies to address the above obstacles becomes a crucial task and remains a further endeavor.

Not long ago, we proposed the concept of “contrary logic” and fabricated different DNA contrary logic pairs (CLP) by using G‐quadruplex DNAzyme, DNA nanoclusters, upconversion nanoparticles, and other nanomaterials as basic elements.^[^ ^21^, ^22^, ^23^, ^24^, ^25^ ^]^ Specifically, two logic gates with opposite functions (CLP = positive/negative) can be implemented in parallel via the same DNA reaction, leading to markedly decreased cost/complexity and elevated computing efficiency. It's worth mentioning that logical recognition of cancer biomarkers was achieved by exploiting the “positive/negative cross‐verification effect” of CLP's dual‐output toward analytical results based on the DNA Cu‐nanocluster platform,^[^ ^22^ ^]^ offering strengthened reliability and accuracy. Nevertheless, to the best of our knowledge, contrary logic has not been combined with isothermal amplification techniques, and the unsatisfactory sensitivity restricted corresponding availability in the field of medical diagnosis to some extent. Under the above circumstances, the merits/demerits of the current CLP and WISO circuit motivated us to design novel programmable WISO DNA nanodevices with maneuverable contrary logic responses, and further explore their diversified application scenarios in information‐processing and molecular diagnostics.

**Scheme 1:**
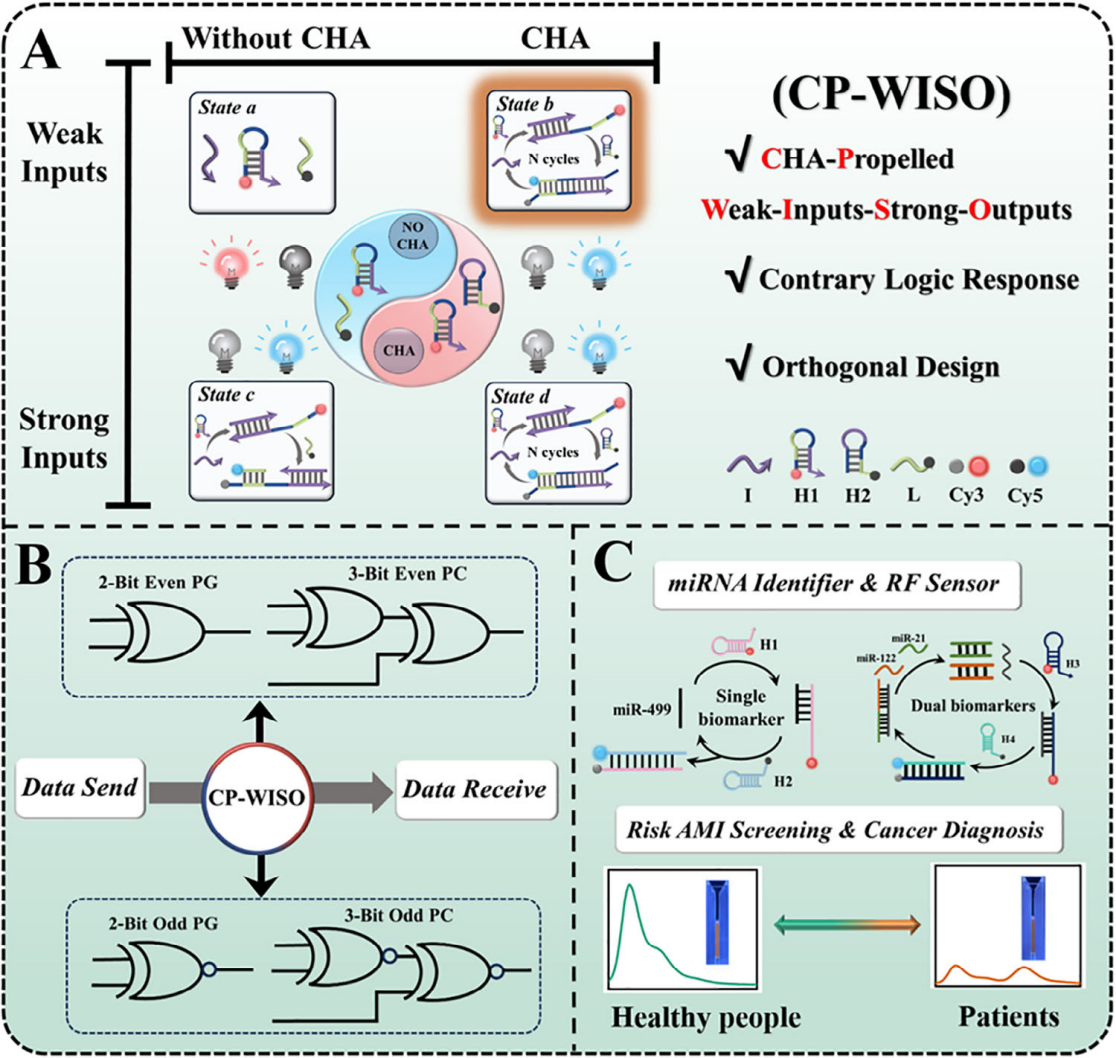
Schematic illustration of the CP‐WISO DNA logic platform with orthogonal design and contrary logic responses and its biosensing applications.

In this work, by harnessing catalytic‐hairpin‐assembly (CHA)‐propelled FRET effect between the donor (Cy3‐DNA) and acceptor (Cy5‐DNA),^[^ ^26^ ^]^ we for the first time constructed a universal CP‐WISO DNA platform with the contrary logic response, **Scheme** **1**A. Surprisingly, the one‐step CHA amplification avoided DNAs’ over‐consumption, complexity, and high cost, and this platform exhibited manipulated orthogonal changes under the trigger of rational inputs. A library of CP‐WISO DNA logic nanodevices with distinct functions (YES∧NOT, OR∧NOR, INH∧IMP, XOR∧XNOR) were exquisitely realized, bringing about satisfactory flexibility, maneuverability, and computing proficiency. Moreover, the even/odd 2‐bit parity generators and 3‐bit parity checkers (pG/pC) were further fabricated (Scheme 1B) in parallel based on the “XOR∧XNOR”‐type CP‐WISO nanodevice, ensuring the normal molecular data transmission. Furthermore, taking miRNA‐499, miRNA‐21, and miRNA‐122 as alternative model targets, the “YES∧NOT” and “AND∧NAND” CP‐WISO nanodevices (working as miRNA identifiers) realized intelligent recognition and ratiometric fluorescent detection of miRNAs in human serums, Scheme 1C, the high reliability, sensitivity, and selectivity provided a reliable toolbox for the early diagnostics of acute myocardial infarction and liver cancer.

## 2 Results and Discussions

### 2.1 Operation Principle of the CP‐WISO System

The detailed operating mechanism of CP‐WISO DNA contrary logic system was illustrated in **Scheme** 1, which demonstrated apparent orthogonal presentation. To realize the weak‐inputs‐strong‐outputs effect, CHA was applied as the representative amplification technique. The weak/strong inputs and with/without the participation of CHA were alternatively combined into 4 different states (a, b, c, d) during the orthogonal design. Notably, 15 and 100 nm DNAs were generally defined as the weak and strong inputs for all CP‐WISO logic devices, respectively. To demonstrate it more clearly, for the DNA reactions assisted with CHA (States b, d), two hairpins (H1, H2) that worked as the basic elements of CHA were assigned as the initial platform, in which the 5′ end of H1 was modified with Cy3 (red and grey balls), and 3′ end of H2 was labeled with Cy5 (blue and black balls). By contrast, for the reactions without CHA (States a, c), H1 and L strands acted as the platform, where the 3′ terminals of L was attached with Cy5. The high/low fluorescence signals of Cy3 at 567 nm and that of Cy5 at 667 nm were taken as dual‐output “1, 0” of contrary logic nanodevices, respectively, and normalized fluorescence intensity 0.50 was set as the universal threshold value. As can be speculated, the occurrence and non‐occurrence of FRET phenomena that are triggered by controllable inputs will generate outputs “0, 1” and “1, 0,” respectively. To corroborate the superiority and powerful operation of CP‐WISO circuit, the ideal circumstances can be interpreted as follows. For the DNA reactions triggered by weak inputs, we could only get the CP‐WISO DNA nanodevices with specific contrary logic responses in the presence of CHA; for that initiated by strong inputs, the CP‐WISO DNA contrary logic nanodevices can be alternatively achieved regardless of the participants of CHA. Moreover, the DNA pG/pC and miRNA identifiers were further fabricated based on the universal CP‐WISO system, their corresponding operation will be thoroughly described in subsequent sections.

### 2.2 YES∧NOT‐Type CP‐WISO DNA Nanodevice

**Figure 1:**
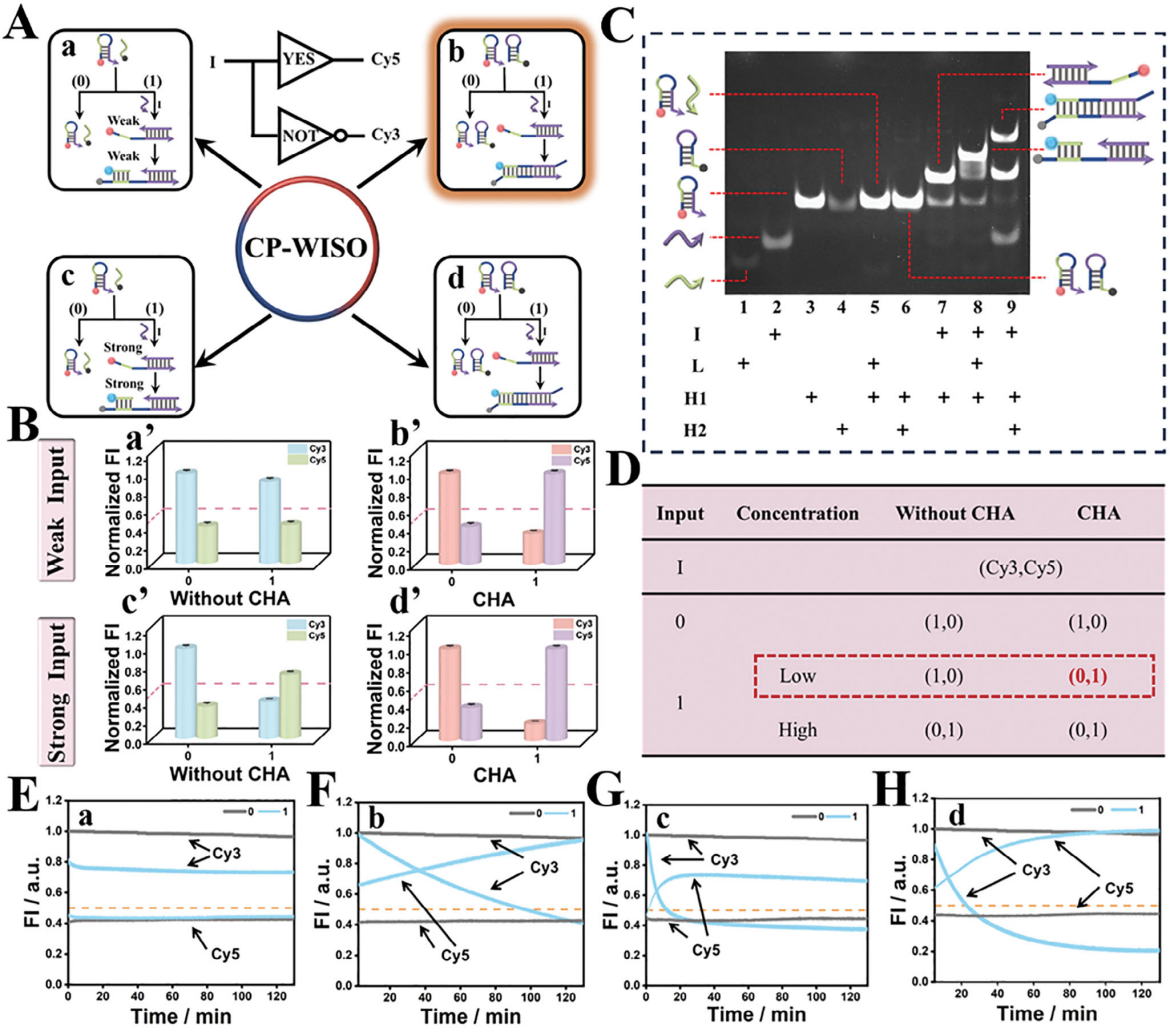
A) Four different inputs of the YES∧NOT‐type CP‐WISO DNA nanodevice. State a (weak‐input & No CHA); State b (weak‐input & CHA); State c (strong‐input & No CHA); State d (strong‐input & CHA). 0 and 1 represent the absence of I and the presence of I, respectively. Inset: Equivalent logic symbol of YES∧NOT contrary logic pair (CLP); B) Corresponding normalized fluorescence column bars of Cy3 and Cy5 under different states. State a’ (weak‐input & No CHA); State b’ (weak‐input & CHA); State c’ (strong‐input & No CHA); State d’ (strong‐input & CHA). The red dashed line shows the threshold value of 0.5; All the error bars were obtained via three independent experiments. C) Verification of DNA hybridization by PAGE gel analysis. Lanes 1–9: L, I, H1, H2, H1+L, H1+H2, H1+I, H1+L+I, H1+H2+I. D) The truth table of the YES∧NOT‐type CP‐WISO DNA nanodevice. E–H) The kinetic fluorescence changes of Cy3 and Cy5 probes under different orthogonal states.

The operation of YES∧NOT‐type CP‐WISO DNA nanodevice (**Figure** **1**A) was analogous to the reactions shown in Scheme 1A, in which strand I that could open H1 and initiate CHA acted as the solely input. Before the operation of logic devices, the temperature, pH, and reaction time were all optimized accordingly by using FI_667_/FI_567_ as the standard reference, Figure S1 (Supporting Information). The best conditions were selected as 37 °C, pH 7.4, 2 h, respectively. For State a (weak‐input & No CHA), the absence and presence of weak I will not cause observable influence on H1, and we could obtain large amounts of strands H1 and L, and few amounts of triplex I/H/L (input = 1), accompanied with no evident FRET. Therefore, the dual‐output will maintain the original “1, 0” no matter weak I is present or not, **Figure** 1B‐a. However, for State b (weak input & CHA), the input‐output correlation will be different. In the absence of weak I, the output is still “1, 0”; in the presence of weak I, I will interact with H1 and H2 consecutively and initiate CHA, resulting in remarkable FRET and corresponding dual‐output “0, 1,” Figure 1B‐b. For States c & d that are triggered by strong I, the absence of strong input will not change the original dual‐output “1, 0.” Whereas, the presence of strong I will induce its interaction with H1 to form intermediate I/H1, which will react with L (State c) or H2 (State d), bringing about the FRET effect and changing the dual‐output into “0, 1”, Figure 1B‐c,d. Corresponding verifications of the above DNA hybridization are given in Figure 1C. Through comparing the bands that appeared in Lane 7–9 and Lane 1–6, the new bands located at higher positions adequately confirmed the formation of I/H1, I/H1/L, and H1/H2 complexes, respectively. For State b (weak‐input & CHA), the input‐output mapping pattern under different input states aligned well with the logic regulation of YES∧NOT CLP (Figure 1D), which adequately proved the successful operation of YES∧NOT‐type CP‐WISO DNA nanodevice. Corresponding fluorescence spectra of the above 4 states were given in Figures S2 and S3 (Supporting Information), respectively. Besides, to further validate the kinetic changes of the concurrent dual‐output, the real‐time normalized fluorescence intensities of Cy3 and Cy5 were monitored. As can be seen in Figure 1E–H, only CHA‐participated states (State b and d) could yield considerable signal‐to‐noise ratios within 80 and 120 min, respectively. To save the cost and reduce the consumption of DNAs, CHA‐participated DNA reactions and 120 min were selected as the desired condition for the CP‐WISO logic devices. The general orthogonal design endowed DNA computing with more powerful flexibility and maneuverability. Moreover, the stability of CP‐WISO DNA nanodevice was further studied by using the same strands that were stored at 4 °C for ≈3 months. As shown in Figure S4 (Supporting Information), the normalized fluorescence outputs of the YES∧NOT logic device just presented <10% decrease (the highest original outputs were taken as 1.0), indicating the excellent stability and repeatability of the universal system.

### 2.3 OR∧NOR‐type CP‐WISO DNA Nanodevice

**Figure 2:**
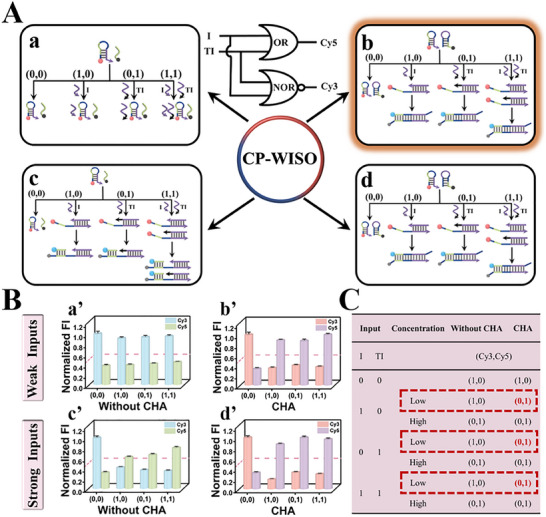
A) Four different states of the OR∧NOR‐type CP‐WISO DNA nanodevice. State a (weak‐input & No CHA); State b (weak‐input & CHA); State c (strong‐input & No CHA); State d (strong‐input & CHA). (0,0) represents the absence of I and TI; for others ditto. Inset: Equivalent logic symbol of OR∧NOR CLP. B) Fluorescent column bars of Cy3 and Cy5 under different input combinations of corresponding states. State a’ (weak‐input & No CHA); State b’ (weak‐input & CHA); State c’ (strong‐input & No CHA); State d’ (strong‐input & CHA). The red dashed line shows the threshold value of 0.5. All the error bars were obtained via three independent experiments. C) Truth table of the OR∧NOR CLP.

For the operation of OR∧NOR‐type CP‐WISO DNA nanodevice, another strand TI was introduced and used as the second input. TI is 5T bases longer than I at its 3′ end and possesses the same function as I. The 4 states of OR∧NOR‐type CP‐WISO DNA nanodevice were vividly depicted in **Figures** **2**A and S5 (Supporting Information) (detailed DNA reactions). For State a (weak‐input & No CHA), the absence and presence of weak I or weak TI will not cause an obvious influence on H1, and we could get large amounts of strands H1 and L, and few amounts of triplex I/H/L or TI/H/L (Figure S5, Supporting Information), accompanied with almost no FRET. Therefore, the dual‐output will keep the initial “1, 0,” regardless of the input combinations (0,0; 0,1; 1,0; 1,1), Figure 2B‐a. While, for State b (weak‐input & CHA), the input‐output patterns will be changed. In the presence of either weak I or weak TI (Input combinations = 0,1; 1,0 or 1,1), I or TI will hybridize with H1 and H2 via CHA, propelling the generation of amplified FRET signals, changing the output into “0, 1.” And only the co‐absence of weak I and TI will not affect the dual‐output “1, 0,” Figure 2B‐b. For States c & d that are triggered by strong I or TI, the presence of anyone of them (Input combinations = 0,1; 1,0 or 1,1) will produce the intermediate I/H1 or TI/H1, and evoke efficient FRET after their interaction with L (State c) or H2 (State d), exporting the dual‐output “0, 1.” Moreover, only the co‐absence of strong I and TI will maintain the dual‐output “1, 0,” Figure 2B‐c,d. Corresponding fluorescence spectra of the above 4 states and the real‐time normalized fluorescence intensity changes of Cy3 and Cy5 were shown in Figures S6–S8 (Supporting Information), respectively. For State b (weak‐input & CHA), the input‐output mapping correlations under different input combinations were in good accordance with the truth table of OR∧NOR CLP (Figure 2C), fully identifying the rational construction of OR∧NOR‐type CP‐WISO DNA nanodevice.

### 2.4 INH∧IMP‐type CP‐WISO DNA Nanodevice

**Figure 3:**
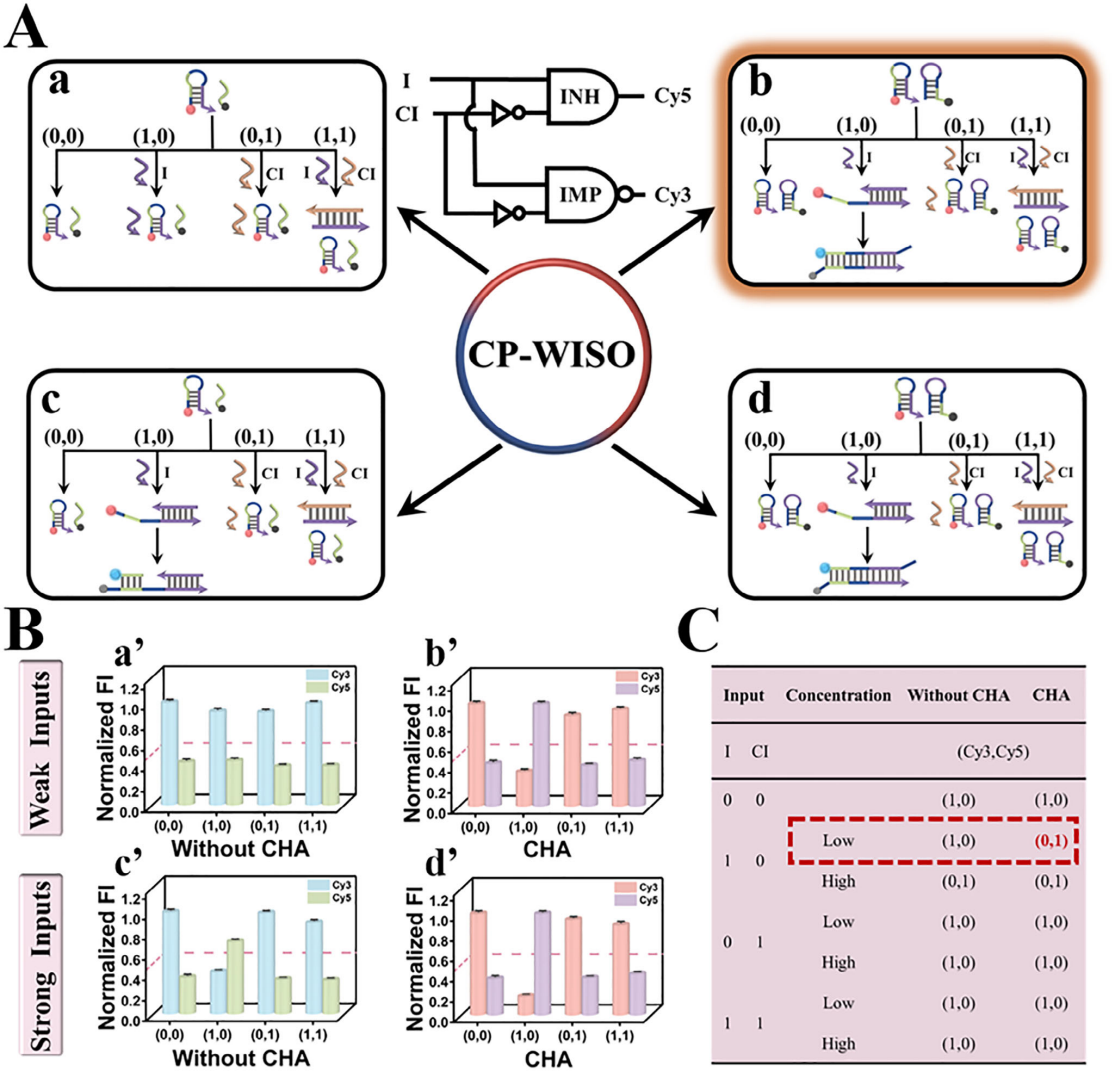
A) Four different states of the INH∧IMP‐type CP‐WISO DNA nanodevice. State a (weak‐input & No CHA); State b (weak‐input & CHA); State c (strong‐input & No CHA); State d (strong‐input & CHA). (0,0) represents the absence of I and CI; others ditto. Inset: Equivalent logic symbol of INH∧IMP CLP. B) Fluorescent column bars of Cy3 and Cy5 under different input combinations of corresponding states. State a’ (weak‐input & No CHA); State b’ (weak‐input & CHA); State c’ (strong‐input & No CHA); State d’ (strong‐input & CHA). The red dashed line shows the threshold value of 0.5. All the error bars were obtained via three independent experiments. C) Truth table of the INH∧IMP CLP.

Different from OR∧NOR‐type CP‐WISO DNA nanodevice, the operation of INH∧IMP one relied on the inhibitory reaction between input strands I and CI, wherein CI is the full complementary strand of I. Analogously, the 4 states of INH∧IMP‐type CP‐WISO DNA nanodevice were clearly illustrated in **Figures** **3**A and S9 (Supporting Information). Corresponding DNA reactions have been verified by PAGE experiments, Figure S10 (Supporting Information). For State a (weak‐input & No CHA), the absence and presence of weak I or weak CI will not bring a noticeable influence on H1, and we could get high concentrations of strands H1 and L, and low ones of duplex I/CI or triplex I/H/L, all of which will not induce efficient FRET. Hence, the dual‐output will keep the original “1, 0,” no matter how both inputs are combined (0,0; 0,1; 1,0; 1,1), Figure 3B‐a. However, for State b (weak input & CHA), the input‐output circumstances will be significantly altered. Only the concurrent presence of weak I and absence of weak CI (Input combination = 1, 0), the weak I will react with H1 and H2 via CHA, generating the amplified FRET signals, transforming the dual‐output into “0, 1.” Besides, during the co‐absence of both inputs (Input combination = 0,0) or the presence of weak CI (Input combination = 0, 1 or 1, 1), we could obtain large amounts of H1 and H2, and few amounts of CI or I/CI duplex, all of which will not bring about effective FRET, yielding dual‐output “1,0,” Figure 3B‐b. For States c & d that are stimulated by strong I or CI, the participation of CHA will not influence corresponding input‐output changes. In the co‐absence of both inputs, the non‐occurrence of FRET will not change the initial dual‐output “1,0.” Besides, in the absence of I and the presence of CI (Input combination = 0, 1), the strong CI will neither interact with the mixture of H1 and L, nor with that of H1 and H2, the FRET still could not take place, maintaining dual‐output “1, 0.” By contrast, in the absence of CI and presence of I (Input combination = 1, 0), I will open H1 to form I/H1 intermediate, which could react with L or H2, triggering obvious FRET and altered dual‐output “0, 1.” Finally, during the coexistence of both inputs (Input combination = 1, 1), I will presumably hybridize with CI to form I/CI duplex and inhibit subsequent CHA amplification and FRET, generating dual‐output “1, 0,” Figure 3B‐c,d. Corresponding fluorescence spectra of these 4 states and the real‐time normalized fluorescence intensity changes of Cy3 and Cy5 were presented in Figures S11–S13 (Supporting Information), respectively. For State b (weak‐input & CHA), the input‐output patterns under different input combinations aligned well with the truth table of INH∧IMP CLP (Figure 3C), corroborating the reasonable operation of INH∧IMP‐type CP‐WISO DNA nanodevice.

### 2.5 XOR∧XNOR‐Type CP‐WISO DNA Nanodevice

**Figure 4:**
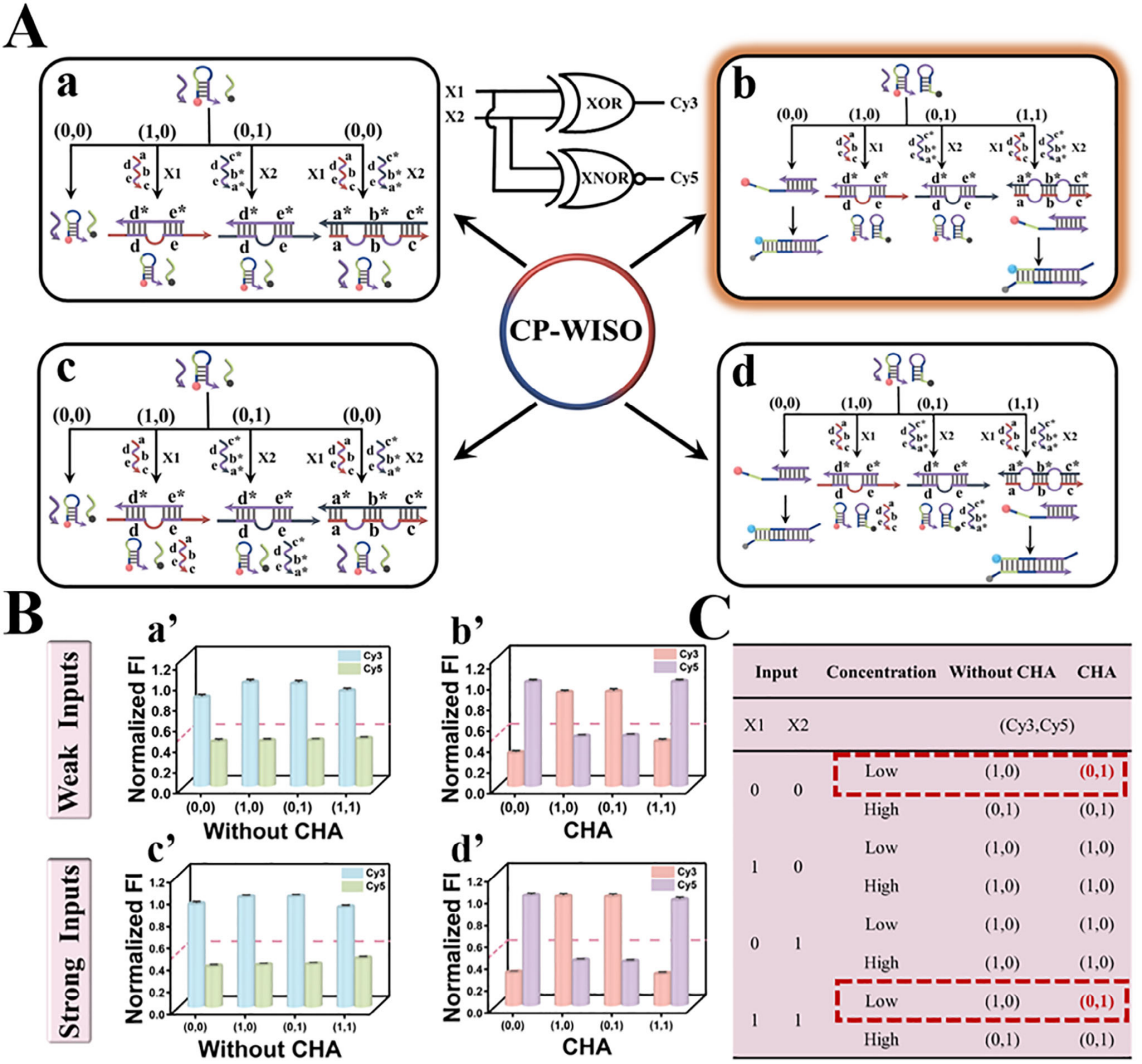
A) Four different states of the XOR∧XNOR‐type CP‐WISO DNA nanodevice. State a (weak‐input & No CHA); State b (weak‐input & CHA); State c (strong‐input & No CHA); State d (strong‐input & CHA). (0,0) represents the absence of X1 and X2; others ditto. Inset: Equivalent logic symbol of XOR∧XNOR CLP. B) Fluorescent column bars of Cy3 and Cy5 under different input combinations of corresponding states. State a’ (weak‐input & No CHA); State b’ (weak‐input & CHA); State c’ (strong‐input & No CHA); State d’ (strong‐input & CHA). The red dashed line shows the threshold value of 0.5. All the error bars were obtained via three independent experiments. C) Truth table of the XOR∧XNOR CLP.

Unlike simple logic gates, XOR and XNOR could execute desired addition/subtraction calculations via their integration with AND (half‐adder) or INH (half‐subtractor) gates.^[^ ^27^, ^28^, ^29^ ^]^ More difficult than the above three CP‐WISO DNA nanodevices, the operation of XOR∧XNOR‐type required the subtle design of DNA strands (**Figure** **4**A; Figure S14, Supporting Information), in which strands X1 and X2 that could prehybridize with I worked as two inputs. Strand X1, it's composed of “a‐d‐b‐e‐c” parts (from 5′ to 3′ end), wherein the d‐e sequences could hybridize with e*‐d* parts of strand I. Similarly, strand X2 is composed of “c*‐d‐b*‐e‐a*” sections (from 5′ to 3′ end), in which the d‐e sequences are complementary to e*‐d* parts of strand I. Apparently, X1 and X2 could also hybridize with each other to form a more stable a/a*‐b/b*‐c/c* partial duplex, and the double d‐e sequences will be looped out. The DNA reactions among strand I, X1 and X2 have been solidly confirmed by corresponding PAGE verifications in Figure S10 (Supporting Information). It should be noted that the concentration of I was kept “weak” throughout the operation of XOR∧XNOR‐type CP‐WISO nanodevice.

For State a (weak‐input & No CHA), the concentrations of X1 and X2 were just the same as that of strand I. No matter weak X1 or weak X2 was present or not, the residual weak I would not induce noticeable changes, and we could get high concentrations of strands H1 and L, and low ones of duplex X1/I/ or X2/I, or X1/X2 or triplex I/H/L, all the products will not trigger significant FRET, keeping the original dual‐output “1, 0,” Figure 4B‐a. However, for State b (weak input & CHA), the input‐output correlations will be different. During the co‐absence of weak X1 and weak X2 (Input combination = 0, 0), the free weak I will interact with H1 and H2 and initiate CHA amplification, resulting in the FRET effect and the changed dual‐output “0,1.” In the presence of any individual input (Input combination = 0, 1 or 1,0), the weak X1 or X2 will prehybridize with I to form weak X1/I or X2/I duplex, the residual large amounts of H1 and H2 indicating the inhibition of CHA and FRET, exporting dual‐output “1,0.” Moreover, during the co‐existence of both inputs (Input combination = 1,1), X1 and X2 preferred to hybridize with each other to form a more stable X1/X2 duplex with few amounts, and the free weak I could still trigger CHA and FRET phenomenon, producing dual‐output “0,1,” Figure 4B‐b.

For State c (strong‐input & No CHA), the strong X1 or X2 will neither react with H1 nor L due to the non‐hybridization between them. Therefore, the presence of weak I will not induce obvious changes, and we could get high concentrations of H1 and L, or X1, X2, and few amounts of X1/I or X2/I duplex and I/H1/L triplex depending on the varied input combinations. The weak FRET effect induced by few amounts of I/H1/L triplex will not cause noticeable dual‐signal changes, maintaining the dual‐output “1,0”, Figure 4B‐c. Finally, for State d (strong‐input & CHA), the co‐absence and co‐existence of strong X1 and X2 will lead to similar DNA reactions to that of State b, and the redundant weak I will initiate efficient CHA and FRET, yielding the dual‐output “0,1.” During the presence of any individual input (Input combination = 0,1 or 1,0), through DNA hybridization, we could get large amounts of H1 and H2, or X1, X2, and low concentrations of X1/I or X2/I duplex based on the different input combinations. The nonoccurrence of the FRET effect resulted in the corresponding dual‐output “1,0,” Figure 4B‐d. Corresponding fluorescence spectra of above 4 states were presented in Figures S15 and S16 (Supporting Information). For State b (weak‐input & CHA), the input‐output patterns demonstrated good accordance with the truth table of XOR∧XNOR CP‐WISO DNA nanodevice (Figure 4C), identifying its rational construction.

### 2.6 Concomitant Operation of Even‐Odd Parity Generator/Checker (pG/PC)

**Figure 5:**
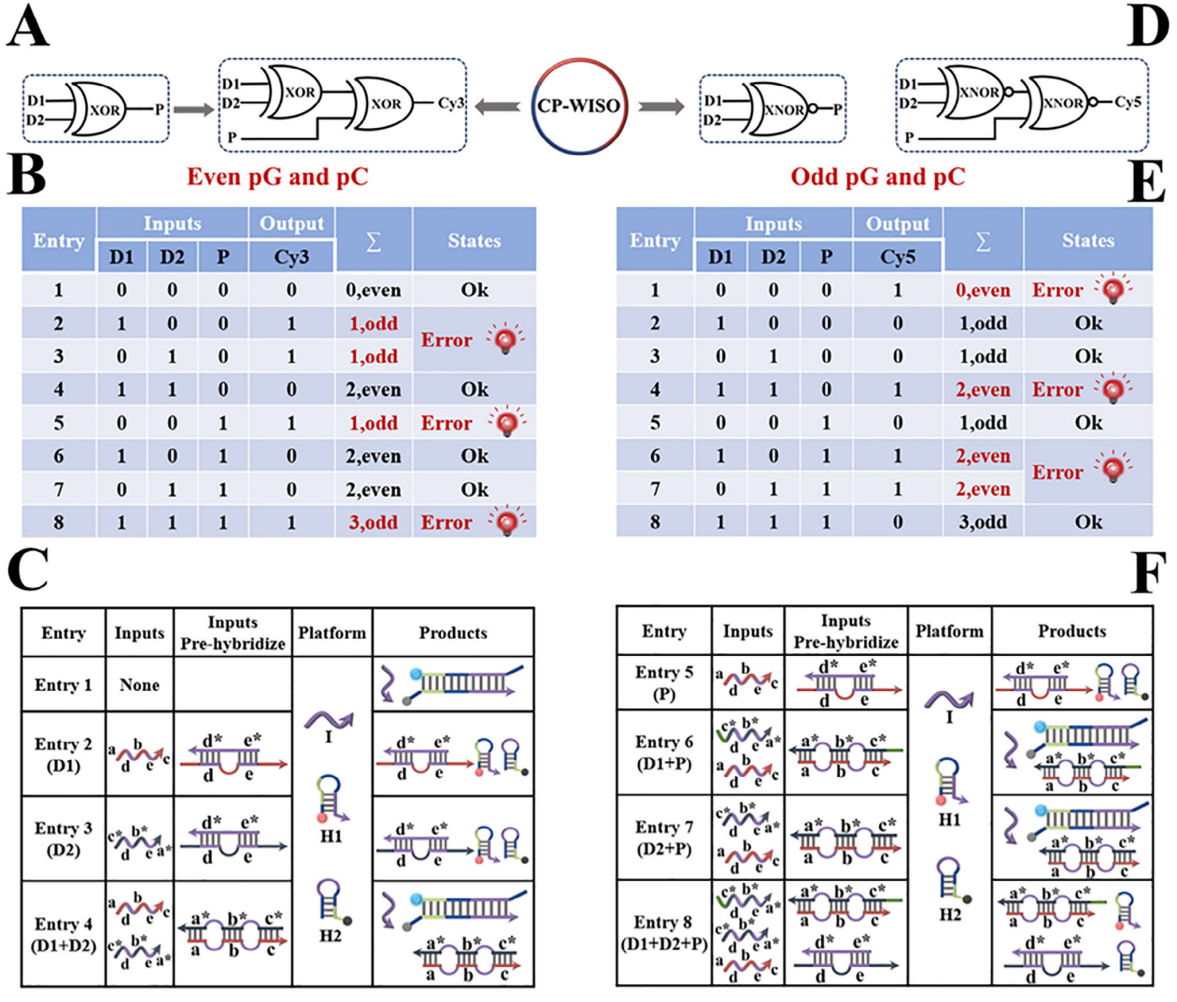
A) Equivalent logic symbol of the 2‐bit even pG and 3‐bit even pC. B) The truth table of the 3‐bit even pC. C) Detailed DNA hybridizations of entries 1–4 for 2‐bit even pG and 3‐bit even pC (under P  =  0 states), D1 = X1, D2 = X2. D) Equivalent logic symbol of the 2‐bit odd pG and 3‐bit odd pC for data transmission. E) The truth table of the 3‐bit odd pC. F) Detailed DNA hybridizations of entries 5–8 for 3‐bit odd pC (under P = 1 states), D1 = X2T, D2 = X2, P = X1.

Apart from the elementary logic pairs for DNA computing, the transmission of binary data also plays a paramount role in information processing.^[^ ^30^ ^]^ The generation of erroneous bits, which usually bring serious effects, is an unavoidable and regular problem. Typically, the occurrence of bit errors can be distinguished by placing a n‐bit parity generator (pG) at the sending end and a (n+1)‐bit parity checker (pC) at the receiving end.^[^ ^31^ ^]^ The pG/pC that originated from XOR or XNOR gates can be classified into even and odd types, wherein the even pG yields an additional parity‐bit P and adds it to initial data string Dn and changing the number of 1′s (∑) into even.^[^ ^32^ ^]^ Taking the delivery of two bits’ data for instance, the 2‐bit even pG will generate the output P through referencing the truth table of an XOR gate. Afterward, the yielded D1D2P data string will be transmitted to the 3‐bit even pC (D1, D2 and P act as three inputs) and analyzed by it (**Figure** **5**A).^[^ ^33^, ^34^ ^]^ During a normal sending, the three bits in D1D2P string will not be influenced and the corresponding ∑ value will keep “even,” accompanied with the output “C = 0” (Normal) that yielded by pC. By contrast, if the transmission is erroneous, the sent bits in D1D2P string will be altered (emergence of bit errors), the ∑ value of the received string will change to “odd” (see Figure 5B), then the pC presents an “alarm” via generating output C = 1. Accordingly, the normal/erroneous data transmission can be facilely detected via pC's outputs. Moreover, the odd pG/pC (Figure 5D,E) demonstrates analogous ability to the even one but relies on the operation of XNOR gates.^[^ ^9^ ^]^

Based on State b of the XOR∧XNOR‐type CP‐WISO DNA nanodevice, we further realized the concomitant operation of even‐odd pG/pC. To demonstrate it more clearly, the construction of 2‐bit even pG and 3‐bit even pC was illustrated as an example, Figure 5A–C. The weak X1 and X2 that functioned as two inputs were introduced to prehybridize with strand I alternatively, and two hairpins (H1 and H2) still worked as the amplification elements. For 2‐bit even pG, the weak X1 and X2 were taken as input D1 and D2, respectively. The FI_567_ values of Cy3 were assigned as the output P of pG. Corresponding DNA reactions under different input combinations were just the same as the above XOR∧XNOR nanodevice. And we could get output P = 1 (No FRET, high FI_567_) in the presence of only one input (input combination = 0,1 or 1,0). Otherwise, the outputs  P =  0 (FRET, low FI_567_) will be generated during the co‐existence and co‐absence of both inputs. For the 3‐bit even pC (P  =  0 states), the operation of Entry 1–4 was identical to 2‐bit even pG. The “low‐high‐high‐low” outputs (C = 0, 1, 1, 0) under varied input combinations corresponded to the data transmission of “normal‐error‐error‐normal” states, respectively.

Particularly, for the 3‐bit even pC (P = 1 states), the input definition of D1D2P string was re‐assigned according to previous reports. ^31^ A new strand X2T, which is 5T bases longer than X2 at the 3′ end, was introduced into the system and functioned as another input, Figure 5F. For Entry 5–8, strands X1, X2T, and the original X2 were applied as input P, D1, and D2, respectively. The detailed operation of 5–8 entries were presented as follows. For Entry 5, the presence of weak X1 will prohibit CHA amplification and FRET effect, yielding the high FI_567_ signal of Cy3 (Output C = 1); for Entry 6 and 7, the coexistence of weak X1 and X2T, or that of weak X1 and X2 will induce the production of X1/X2T or X1/X2 duplex, initiating subsequent CHA reaction and evident FRET, producing low FI_567_ signals of Cy3 (Output C = 0); for Entry 8, three strands will be present at the same time, the weak X1 could hybridize with weak X2 (or X2T) with the same concentration, and the redundant weak X2T (or X2) could hybridize with weak I, which will not trigger the CHA reaction and subsequent FRET phenomenon, generating the high FI_567_ signal of Cy3 (Output C = 1). Accordingly, the overall “high‐low‐low‐high” outputs under different input combinations represented “error‐normal‐normal‐error” states during data transmission, respectively. The fluorescence responses of different entries (Figure S17A, Supporting Information) aligned well with the truth table in Figure 5B.

Additionally, for the 2‐bit odd pG and 3‐bit odd pC (Figure 5D,E), the DNA reactions of all entries were just the same as those of even ones, yet the FI_667_ values of Cy5 were identified as corresponding outputs. Learning from the truth table (Figure 5E), the input‐output patterns were completely opposite to those of even ones. Corresponding outputs produced by different entries of the odd pC were shown in Figure S17B (Supporting Information). To the best of our knowledge, it's the first time that the even‐odd pG/pC was concurrently realized on a universal platform without additional data re‐processing or bit‐conversion. The subtle design could greatly meet the requirements of variability, security, and adjustability of data string under various circumstances.

### 2.7 miRNAs Identifiers and Ratiometric Fluorescent Sensors

To meet the emerging needs in this booming era, a versatile DNA logic system should not only execute specific molecular computing functions and information‐processing but also be expected to present surprising power in biosensing and medical diagnostics.^[^ ^35^, ^36^, ^37^, ^38^, ^39^, ^40^, ^41^ ^]^ The CP‐WISO circuits could amplify detection signals and improve the sensitivity, and the contrary logic outputs have been confirmed to perform “positive/negative cross‐verification” toward analytical results. Therefore, the CP‐WISO DNA contrary logic nanodevice that integrated both aspects of advantages will strengthen the reliability and anti‐interference ability while maintaining high sensitivity, which could work as a powerful molecular toolbox for clinical diagnostics. In 2024, the Nobel Prize in Medicine was assigned to two scientists in the area of miRNA due to their excellent work.^[^ ^42^, ^43^ ^]^ As non‐coding short RNA strands, many miRNAs have been confirmed as typical biomarkers for varieties of diseases.^[^ ^44^, ^45^, ^46^, ^47^, ^48^, ^49^, ^50^ ^]^ Acute myocardial infarction (AMI) is a kind of heart disease with high mortality‐rate that badly threatens human life,^[^ ^51^, ^52^ ^]^ making its early warning more and more important. Not long ago, miR‐499 has been broadly identified as the characteristic biomarker for AMI, and researchers established different strategies to detect miR‐499 accordingly.^[^ ^53^, ^54^, ^55^ ^]^ Analogously, miR‐21 and miR‐122 have been confirmed as the valid indicator of liver cancer. The expression level of them was much higher in liver cancer cells (such as HepG2) than that in normal cells.^[^ ^56^, ^57^, ^58^ ^]^ Nevertheless, the accurate identification and sensitive detection of the above miRNAs in complicated human serum samples still faces crucial challenges.

**Figure 6:**
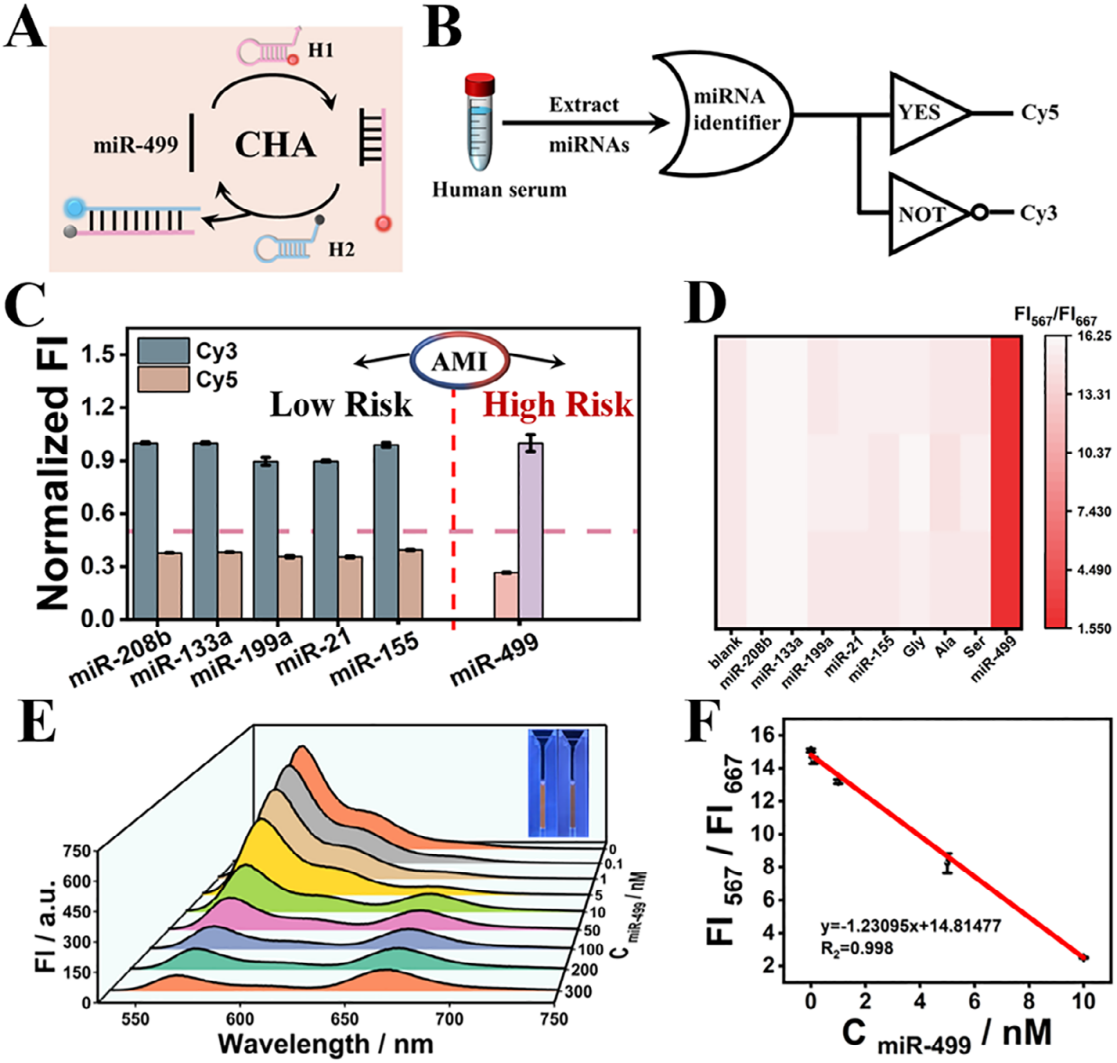
A) Schematic diagram of the CHA reaction for miR‐499 detection. B) Logic symbol of the YES∧NOT‐type CP‐WISO nanodevice for identifying different miRNAs. C) Column bars of normalized fluorescence intensities of Cy3 and Cy5 in the presence of different miRNA inputs. All the error bars were obtained via three independent experiments. D) Corresponding heat maps triggered by different miRNAs. E) The fluorescence waterfall spectra with the addition of various concentrations of miR‐499. (Inset: photographs under UV lamp when the concentration of miR499 is 0 nm (left) and 100 nm (right)); F) Linear relationship between the FI_567_/FI_667_ ratio and the concentrations of miR‐499 from 0.1 to 10 nm. All the error bars were obtained via three independent experiments.

Herein, taking miR‐499 as the initial model target and using the redesigned hairpins H1 and H2 as amplification elements, we fabricated a target‐responsive “YES∧NOT”‐type CP‐WISO nanodevice (**Figure** **6**A,B), which could function as the miRNA identifier. Similarly, the fluorescence signals of Cy3 at 567 nm (FI_567_) and that of Cy5 at 667 nm (FI_667_) were also used as the dual‐output, respectively. As we all know, there are different types of DNAs, miRNAs, and amino acids that pose challenges to the accurate analysis of miR‐499 in real samples. Hence, we used human serums that being diluted with varieties of representative miRNAs for different diseases (miR‐499, ‐208b, ‐133a, ‐199a, ‐21, ‐155, Gly, Ala, Ser) as the complicated biological matrix to explore the feasibility of screening potential risky AMI cases. As presented in Figure 6C, the presence of any non‐target interferential miRNAs will not change the original dual‐output “1, 0” of Cy3/Cy5, corresponding to the “low‐risk” of AMI. When target miR‐499 with pathogenic concentration was introduced, it will interact with H1, H2 and initiate CHA, resulting in satisfactory FRET and the changed dual‐output “0,1”, signifying the “high‐risk” of AMI. Corresponding heat maps of different groups shown in Figure 6D adequately corroborated the excellent selectivity (Figure S18, Supporting Information) and the accuracy of AMI's logical diagnostics. Moreover, the amplified quantitative detection of miR‐499 was further achieved under the assistance of the ratiometric fluorescence technique. Learning from the fluorescent waterfall spectra, Figure 6E, the fluorescence intensity of Cy3 and Cy5 exhibited decreased and increased tendencies with the introduction of miR‐499, respectively. Two good linear relationships between ratiometric values FI_567_/FI_667_ and varied contents of targets (or the logarithmic values) can be obtained (y = ‐1.230x+14.814, R^2^ = 0.998), Figure 6F and Figure S19 (Supporting Information). A LOD of 49 pM can be achieved through careful calculation (S/N = 3). The selectivity and sensitivity of “YES∧NOT”‐type CP‐WISO nanodevice could compete with many previous miRNA biosensors after typical comparison (Table S2, Supporting Information). The relative recovery rate further demonstrated the excellent applicability of the DNA nanodevice in human serum (Table S3, Supporting Information).

**Figure 7:**
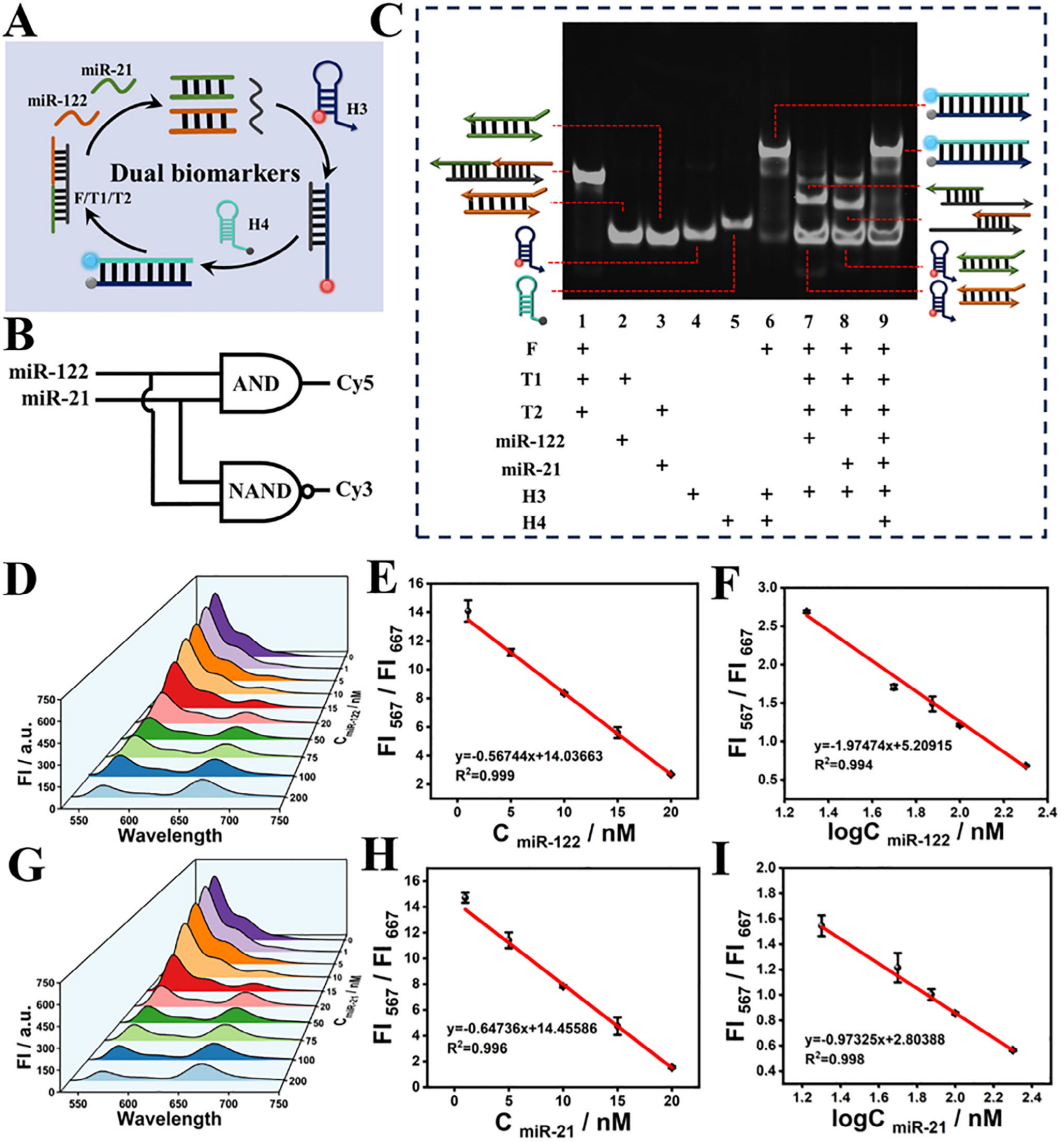
A) Schematic diagram of the CHA reaction for miR‐122 and miR‐21 detection. B) Logic symbol of the AND∧NAND‐type CP‐WISO nanodevice. C) PAGE results to identify the reactions between different DNAs. D) The fluorescence waterfall spectra in the presence of various contents of miR‐122. E,F) The linear relationship between the FI_567_/FI_667_ values and the concentrations of miR‐122, from 0 to 200 nm; G) The fluorescence waterfall spectra in the presence of various contents of miR‐21. H,I) Linear relationship between the FI_567_/FI_667_ values and the contents of miR‐21, from 0 to 200 nm. All the error bars were obtained via three independent experiments.

The effective operation of above miR‐499‐triggered “YES∧NOT”‐type CP‐WISO nanodevice motivated us to explore the potential of above universal system in identifying/detecting dual‐biomarkers and achieving more accurate disease diagnostics. According to previous reports,^[^ ^56^, ^57^, ^58^ ^]^ the level of two miRNAs (miR‐122 and miR‐21) will be evidently changed at the early stage of liver cancer. Subsequently, we further designed an “AND∧NAND”‐type CP‐WISO nanodevice (**Figure** **7**A,B) that was stimulated by above two liver cancer‐related inputs, miR‐122 and miR‐21. Specifically, the triplex F/T1/T2 was introduced to assist the operation of the “AND∧NAND”‐type CP‐WISO nanodevice, in which strand F could partially hybridize with T1 and T2 to form the initial triplex, and T1 and T2 are the full complementary strands of miR‐122 and miR‐21, respectively. The fluorescence signals of Cy3 at 567 nm (FI_567_) and that of Cy5 at 667 nm (FI_667_) were also applied as the dual output, respectively. In the absence of both inputs, the F/T1/T2 triplex could neither interact with H3 and H4, nor initiate the CHA amplification, maintaining the original dual‐output “1, 0” of Cy3/Cy5. Whereas, in the presence of both inputs, T1 and T2 will hybridize with miR‐122 and miR‐21, respectively, and produce duplex T1/miR‐122 and T2/miR‐21, releasing the F strand, which could act as the activator of CHA reaction and generate the altered dual‐output “0, 1,” indicating the high risk of liver cancer. Corresponding DNA reactions have been vividly proved by the PAGE images, Figure 7C, and Figure S20 (Supporting Information). Only the co‐existence of two miRNAs could release strand F and then initiate CHA reaction. Moreover, the amplified quantitative detection of two miRNAs was further alternatively achieved via ratiometric fluorescence (During the detection of one kind of miRNA, another miRNA with suitable content was present). As presented in the fluorescent waterfall spectra, Figure 7D,G, the fluorescence intensity of Cy3 and Cy5 showed decreased and increased tendencies with the addition of miR‐122 or miR‐21, respectively. And excellent linear relationships between FI_567_/FI_667_ values (or the logarithmic ratiometric values) and different concentrations of targets can be realized: y = ‐0.567x+14.037 (R^2^ = 0.999) for miR‐122, Figure 7E,F, and y = ‐0.647x+14.456 (R^2^ = 0.996) for miR‐21, Figure 7H,I, respectively. The LODs of 99.8 pM for miR‐122 and 86 pM for miR‐21 can be calculated (S/N = 3), which were competitive in contrast with many miRNA sensors that propelled via single‐step amplification. The reasonable operation of “AND∧NAND”‐type CP‐WISO nanodevice and its “positive/negative cross‐verification effect” facilitated the accurate discrimination and diagnostics of liver cancer‐related diseases via dual‐channel contrary logic responses.

## 3 Conclusion

In summary, we reported the first catalytic‐hairpin‐assembly (CHA)‐propelled weak‐inputs‐strong‐outputs (CP‐WISO) DNA platform with contrary logic responses. Relying on CHA amplification and the outstanding FRET between Cy3/Cy5 DNA probes, a library of CP‐WISO DNA contrary logic nanodevices with orthogonal design and the same threshold value was accomplished. Several conspicuous merits that distinguish this work from previous ones can be illustrated as follows. First, it not only avoided DNAs’ over‐consumption, complexity, and high cost to a great extent, but also bring about satisfactory flexibility, maneuverability, and computing proficiency. Second, the even and odd parity generators/checkers (pG/pC) were concomitantly fabricated under the support of “XOR∧XNOR”‐type CP‐WISO nanodevice, guaranteeing the efficient discrimination of normal/erroneous bits through molecular data transmission. More surprisingly, by exploiting the “positive/negative cross‐verification effect” of “YES∧NOT” and “AND∧NAND”‐type CP‐WISO nanodevice, we realized intelligent recognition and ratiometric fluorescent detection of miRNA‐499, miRNA‐122, and miRNA‐21 in complicated human serums, offering a vivid prototype for the early diagnostics of acute myocardial infarction and liver cancer. Overall, this work paved unique horizons for the design of novel biocomputing systems and cost‐effective molecular diagnostic toolboxes. By incorporating with CRISPR‐Cas techniques, nanozymes, machine‐learning algorithms, and lateral flow test strips, more and more application scenarios in point‐of‐care analysis, food safety, medical test kits, and environmental monitoring can be promisingly envisioned.

## 4 Experimental Section

### Materials and Reagents

All DNAs and miRNAs were purchased from Sangon Biotech Co., Ltd. (Shanghai, China) and their sequences were listed in Tables S1 (Supporting Information). The oligonucleotides were dissolved in distilled water and quantified according to the extinction coefficients (ε260nm, M^−1^ cm^−1^: A = 15 400, G = 11 500, C = 7400 and T = 8700) by UV–vis absorption spectroscopy (Shimadzu UV‐2600i, China). All experiments were conducted using Tris‐HCl buffer (25 mm Tris, 50 mm KCl, 100 mm NaCl, 20 mm MgCl_2_, pH 7.4). Tris was obtained from Sangon Biotech Co., Ltd. (Shanghai, China); KCl, NaCl, MgCl_2_ were all purchased from Sinopharm Chemical Regent Co. (Shanghai, China). TBE buffer was obtained from Beijing Solarbio Science & Technology Co. Ltd. (Beijing, China). All chemicals were of analytical grade and the water used in the experiments was purified by a Millipore system.

### Apparatus

The fluorescence was measured on a Cary eclipse fluorescence spectrophotometer (Agilent). The excitation wavelength was set as 512 nm. The slit widths for the excitation and emission were 10 and 10 nm, respectively. The emission spectra were collected from 532 to 750 nm. Gel images were pictured by the Tanon MINI Space1000.

### Native Polyacrylamide Gel Electrophoresis (PAGE)

The concentrations of all DNAs used in the PAGE experiment were 1 µm. 10 µL samples containing various DNAs were mixed with 2 µL 6×loading buffer in every lane. Then, the DNA samples were loaded into the 15% polyacrylamide hydrogel for electrophoresis. The electrophoresis was performed under the constant voltage of 60 V for 105 min in 1x TBE buffer. Finally, the gels were photographed by the Tanon MINI Space1000 after staining at room temperature for 7 min with Gel‐red.

### Operation of CP‐WISO DNA Logic Nanodevices

All DNA probes were dissolved in Tris‐HCl buffer. Hairpins H1, H2 were heated to 88 °C for 7 min, slowly cooled down to 37 °C and kept at 37 °C for 2 h before use. Besides, other DNA strands were heated at 88 °C for 7 min and cooled down to room temperature before use. The reaction volume was 300 µL.

For the YES∧NOT‐type CP‐WISO DNA nanodevice, H1, and H2 were assigned as the initial platform of CHA, while H1 and L strands acted as the platform without CHA. The concentrations of all of them were set as 200 nm. Strand I was used as the input, where the concentration of weak input was 15 nm and that of strong input was 100 nm. I of 15 nm or 100 nm were added to two platforms and reacted at 37 °C for 2 h in Tris‐HCl buffer. Then, the fluorescence signals of Cy3 and Cy5 of four states were measured.

For the OR∧NOR‐type CP‐WISO DNA nanodevice, the platforms were the same as the YES∧NOT one mentioned above. I and TI were taken as two inputs. The concentrations of different input combinations were set as 15 nm (weak inputs) and 100 nM (strong inputs), respectively, then were added to two platforms and reacted at 37 °C for 2 h in Tris‐HCl buffer. Finally, the fluorescence intensity of Cy3 and Cy5 of four states was measured. For the INH∧IMP‐type CP‐WISO DNA nanodevice, the platforms were the same as the YES∧NOT one mentioned above. 15 nm I and 15 nm CI were taken as two inputs. Subsequent steps were almost the same.

For the XOR∧XNOR‐type CP‐WISO DNA nanodevice, 15 nm I, 200 nm H1,200 nm H2 and 15 nm I, 200 nm H1, 200 nm L were respectively taken as the new platforms with or without CHA. X1, and X2 were used as two inputs, where the concentrations of weak inputs were still 15 nM and that of the strong inputs were 100 nm. X1, X2, and X1/X2 duplex will hybridize with I for 30 min at 37 °C in advance before their reaction with H1, H2, or H1, L. Subsequent steps were the same as those described above.

For the operation of an even‐odd parity generator/checker (pG/PC), 15 nm I, 200 nm H1, and 200 nm H2 were selected as solely platforms. The 15 nm D1, D2, and P were combined in different ways. Different input combinations were pre‐mixed with 15 nM I. Then, H1 and H2 were added to different DNA mixtures and incubated at 37 °C for 2 h.

### Disease Diagnosis Based on CHA Amplification Strategy and Specificity Analysis

Similarly, Hairpins H1, H2 were heated to 88 °C for 7 min, slowly cooled down to 37 °C and kept at 37 °C for 2 h before use. Then, various concentrations of miR‐499 (or miR‐122 or miR‐21) were introduced into the system containing 200 nm H1,200 nm H2 (or H3 and H4). The final volume of the above solution was adjusted to 300 µL and incubated at 37 °C for 2 h. Subsequently, a series of fluorescence signals were recorded using a fluorescence spectrophotometer. The selectivity was explored by replacing miR‐499 with other miRNAs, in which the concentration of the target was 20 nm and that of non‐targets was 100 nm.

### Applications in Serum Sample

The recovery tests were conducted in human serum, which was diluted 1000 times with Tris‐HCl buffer for further use. Several different concentrations of miR‐499 selected within the linear range were added to the diluted human serum solution containing 200 nm H1 and 200 nm H2. The reaction conditions were consistent with those described above, and then the recovery rates were calculated. In addition, different miRNAs were used to replace miR‐499 and tested in human serum samples.

## Conflict of Interest

The authors declare no conflict of interest.

## Supporting information



Supporting Information

## Data Availability

The data that support the findings of this study are available from the corresponding author upon reasonable request.
