# Supplementary material for: Catalytic Hairpin Assembly‐Propelled Weak‐Inputs‐Strong‐Outputs (CP‐WISO) DNA Logic Nanodevices with Orthogonal Design and Contrary Logic Responses
Source: Adv Sci (Weinh). 2025 Jun 9;12(33):e01430. doi: 10.1002/advs.202501430 (PMC12412575; doi:10.1002/advs.202501430)
Supplement: Supplementary file 1 — Supporting Information [file ADVS-12-e01430-s001.docx]

*Supporting Information*

Catalytic Hairpin Assembly-Propelled Weak-Inputs-Strong-Outputs (CP-WISO) DNA Logic Nanodevices with Orthogonal Design and Contrary Logic Responses

Xujuan Lv,^†^ Jiawen Han,^†^ Juan Wang, Zhihua Lv*, Baojian Huang, Shuai Qin, Xuxin Yan, Shaojun Dong* and Daoqing Fan*

**X. Lv, J. Han, Z. Lv, B. Huang, S. Qin, X. Yan, D. Fan.**

Key Laboratory of Marine Drugs, Ministry of Education, School of Medicine and Pharmacy, Ocean University of China, Qingdao, Shandong, 266003, China; Laboratory for Marine Drugs and Bioproducts, Qingdao Marine Science and Technology Center, Qingdao, Shandong, 266237, China.
E-mail: Lvzhihua@ouc.edu.cn; [fdq9688@ouc.edu.cn; dqfan93@163.com](mailto:fdq9688@ouc.edu.cn;%20dqfan93@163.com).

**S. Dong, D. Fan.**

State Key Laboratory of Electroanalytical Chemistry, Changchun Institute of Applied Chemistry, Chinese Academy of Sciences, Changchun, 130022, China.

E-mail: [dongsj@ciac.ac.cn](mailto:dongsj@ciac.ac.cn);[fdq9688@ouc.edu.cn; dqfan93@163.com](mailto:fdq9688@ouc.edu.cn;%20dqfan93@163.com).

**J. Wang.**

Intelligent Wearable Engineering Research Center of Qingdao, Research Center for Intelligent and Wearable Technology, College of Textiles and Clothing, State Key Laboratory of Bio-Fibers and Eco-Textiles, Qingdao University, Qingdao 266071, China.

^†^ These authors contributed equally to this work.

**Table S1.** Sequences of DNAs and miRNAs used in this work.

| Name | Sequences (5'-3') |
| --- | --- |
| I | TTA AGA CTT GCA GTG ATG TTT |
| L | GAAACAAACATCAC |
| H1 | Cy3-TTG CAG TGA TGT TTG TTT CAG TGC ACA AAA CAT CAC TGC AAG TCT TAA |
| H2 | TTT GTT TCA GTG CAG TGA TGT TTT GTG CAC TGA AAC AAA CAT CAC-Cy5 |
| H3 | Cy3-TTG ATG GAG TGT GTT CAG GAA GAC GTT CAC ACT CCA TCA ACA TCA G |
| H4 | GTT CAG GAA GAC GAG TAG ATG GAG TGT GAA CGT CTT CCT GAA CAC ACT C-Cy5 |
| TI | TTA AGA CTT GCA GTG ATG TTT TTT TT |
| CI | AAA CAT CAC TGC AAG TCT TAA |
| X1 | CAG TAG CAC GAG GTA AAC ATC ACT GCG CTT AGC TGC AAG TCT TAA GGT GTT GGT GAG |
| X2 | CTC ACC AAC ACC AAA CAT CAC TCA GCT AAG CGG CAA GTC TTA AAC CTC GTG CTA CTG |
| F | ATC AGA CTG ATG TTG ATG GAG TGT GAC AAT G |
| T1 | CAA ACA CCA TTG TCA CAC TCC A |
| T2 | TCA ACA TCA GTC TGA TAA GCT A |
| miR-499 | UUA AGA CUU GCA GUG AUG UUU |
| miR-122 | UGG AGU GUG ACA AUG GUG UUU G |
| miR-21 | UAG CUU AUC AGA CUG AUG UUG A |
| miR-199a | CCC AGU GUU CAG ACU ACC UGU UC |
| miR-133a | AGC UGG UAA AAU GGA ACC AAA U |
| miR-208b-5p | AAG CUU UUU GCU CGA AUU AUG U |
| miR-155 | UUA AUG CUA AUC GUG AUA GGG GU |

**
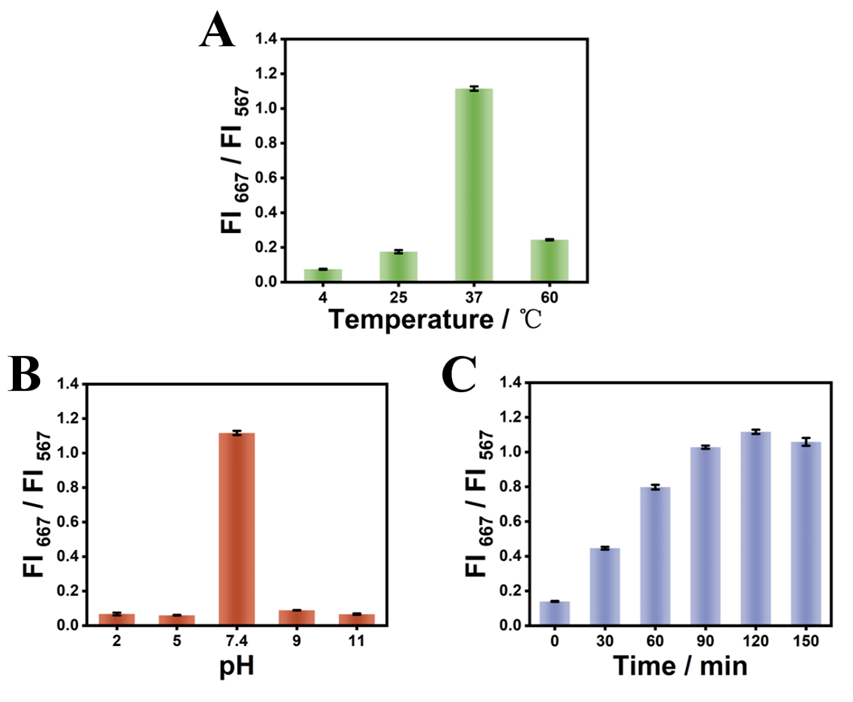
Figure S1.** Optimization experiments of the CP-WISO platform under different (A) temperature. (B) pH and (C) reaction time. All the error bars were obtained via three independent experiments.

**Figure S2. (**A) Equivalent logic symbol of YES∧NOT CLP. (B) Fluorescence spectra in response to state a’. (C) Fluorescence spectra in response to state b’.
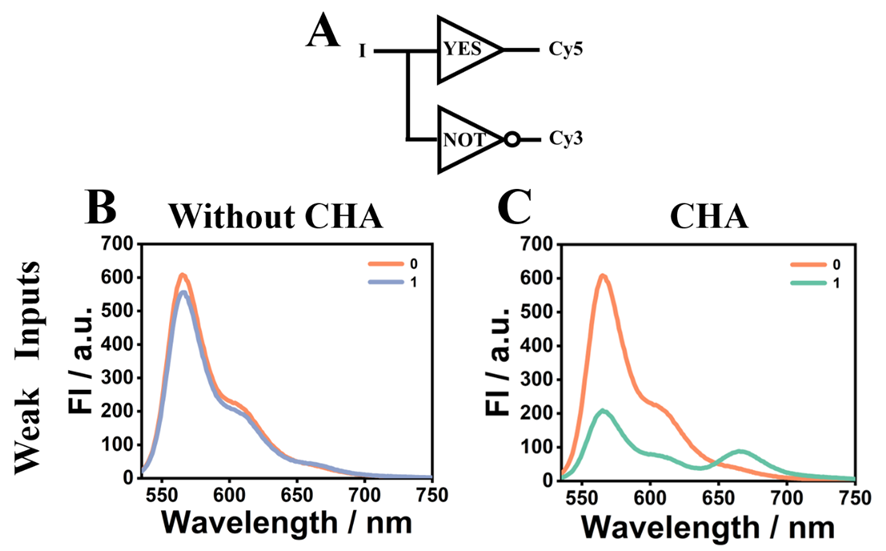


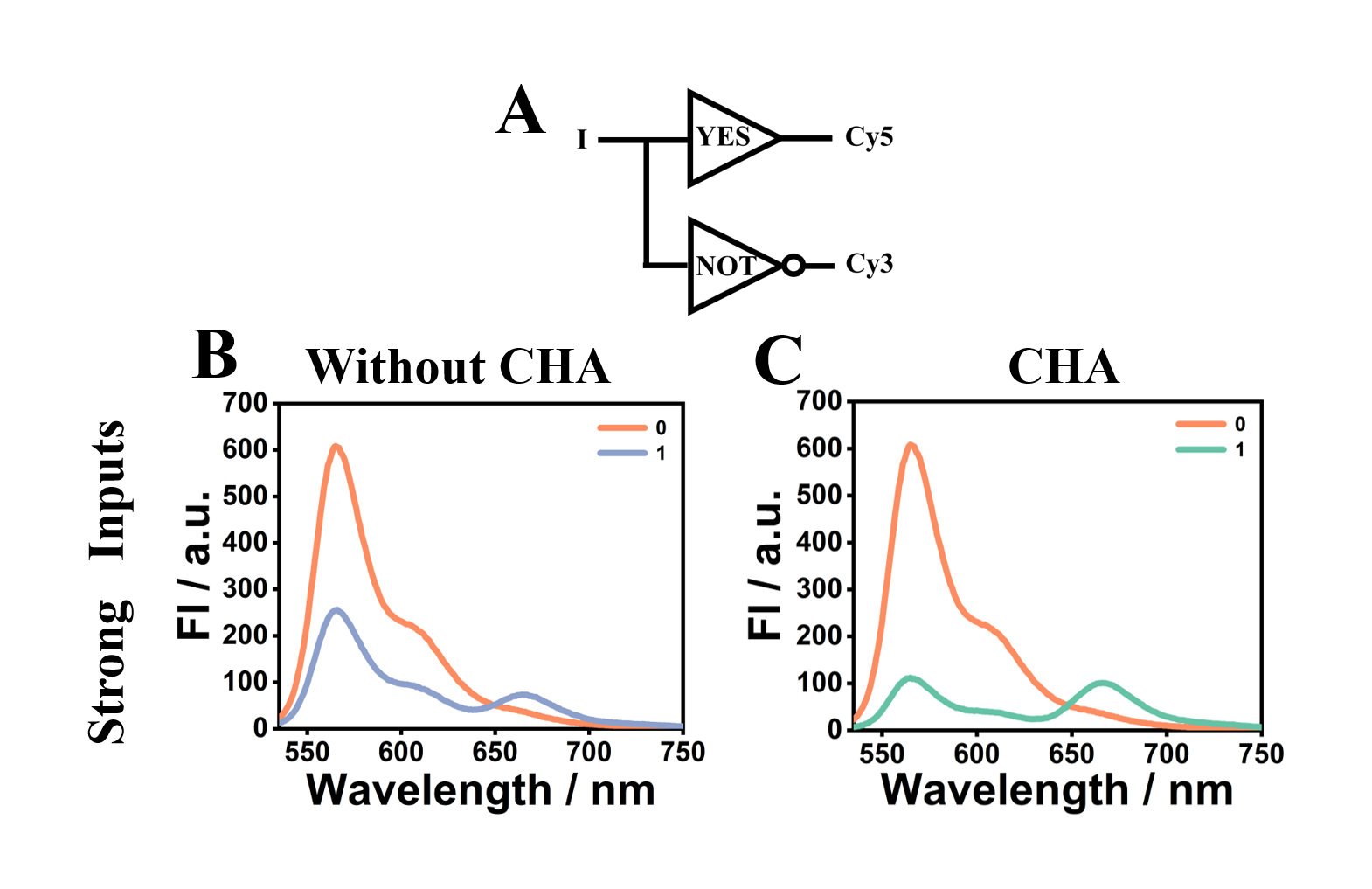
**Figure S3. (**A) Equivalent logic symbol of YES∧NOT CLP. (B) Fluorescence spectra corresponding to state c’. (B) Fluorescence spectra corresponding to state d’.


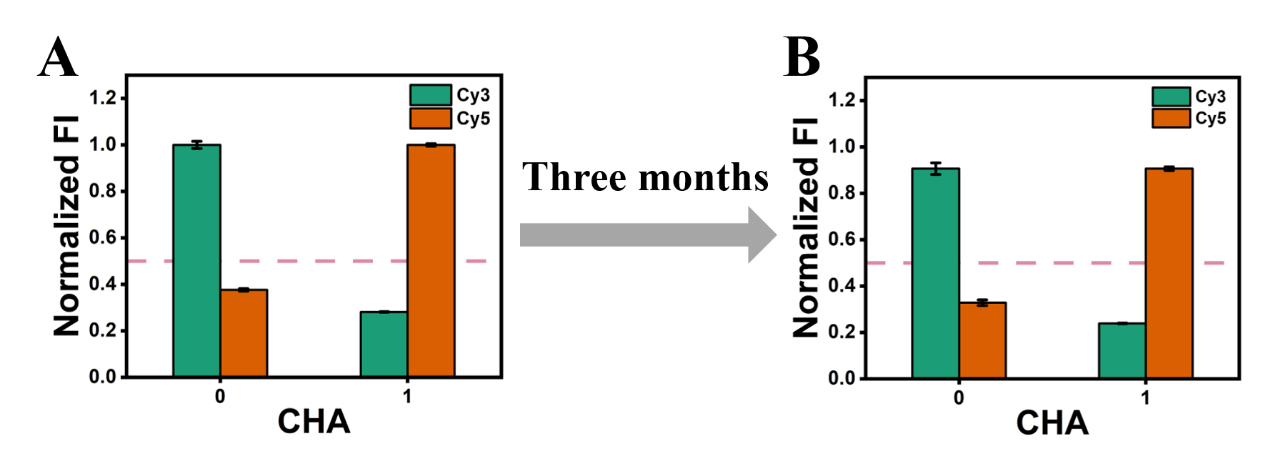


**Figure S4.** Stability of State b of the YES∧NOT CLP before and after 3 months. For (B), the original DNA strands were stored at 4°C for about 3 months. All the error bars were obtained via three independent experiments.


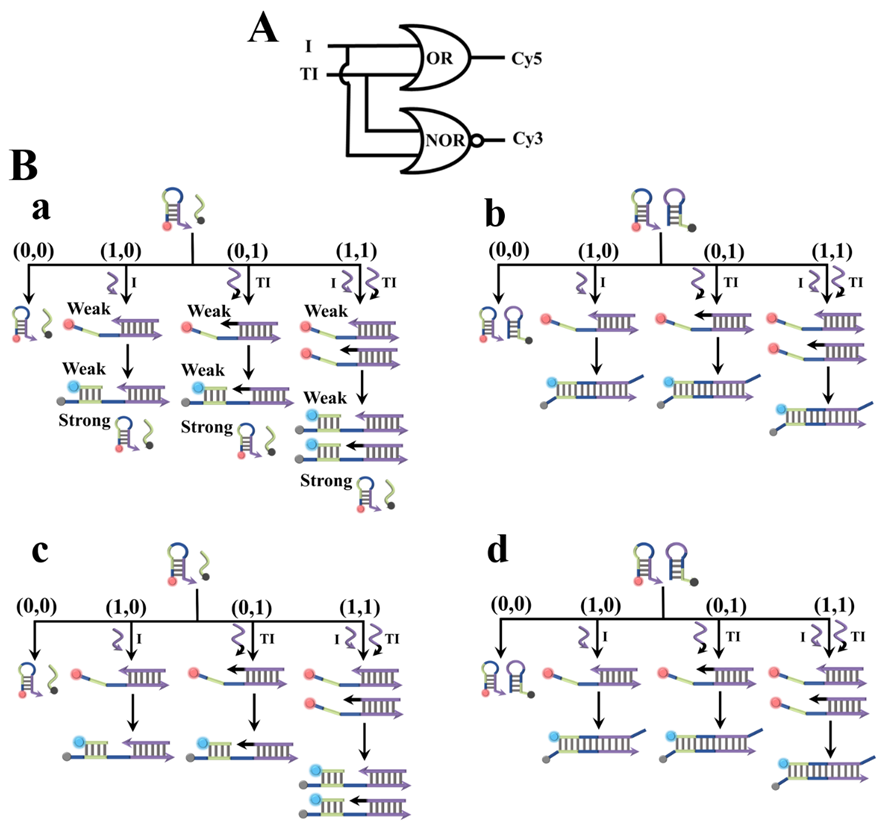
**Figure S5. (**A) Equivalent logic symbol of OR∧NOR CLP. **(**B) Detailed DNA reactions under different input combinations in four states. State a (weak-input & No CHA); State b (weak-input & CHA); State c (strong-input & No CHA); State d (strong-input & CHA).


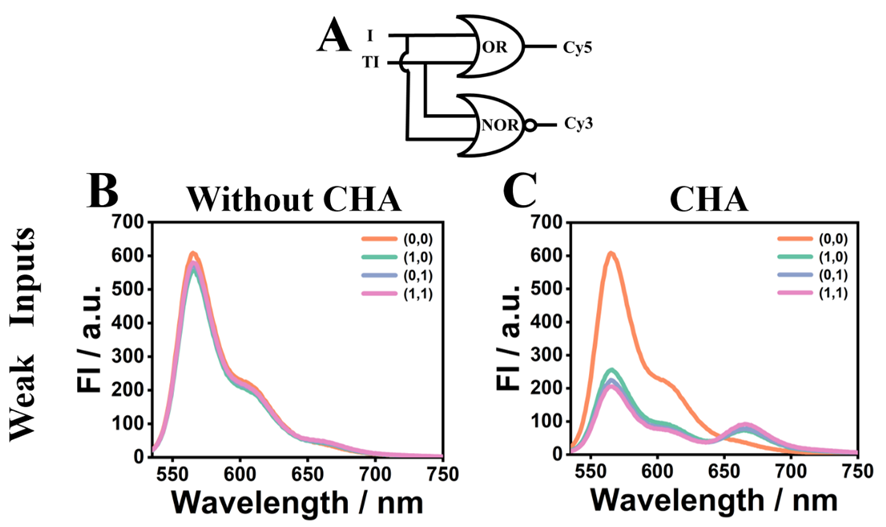
**Figure S6. (**A) Equivalent logic symbol of OR∧NOR CLP. (B) Fluorescence spectra corresponding to state a’. (B) Fluorescence spectra corresponding to state b’.


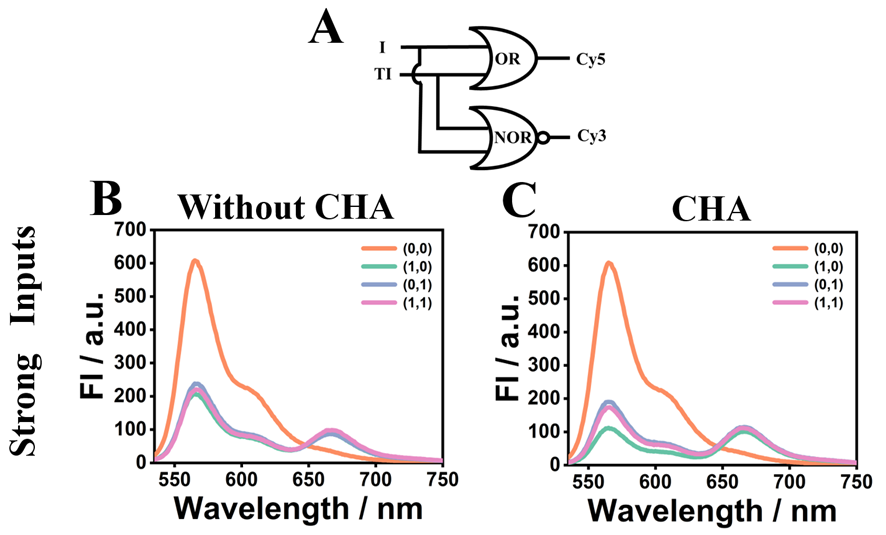
**Figure S7. (**A) Equivalent logic symbol of OR∧NOR CLP. (B) Fluorescence spectra corresponding to state c’. (B) Fluorescence spectra corresponding to state d’.

**Figure S8.** The kinetic fluorescence changes of Cy3 and Cy5 of the OR∧NOR CLP under 4 orthogonal states. (A) weak input & No CHA, (B) weak input &CHA, (C) strong input & No CHA, (D) strong input & CHA.
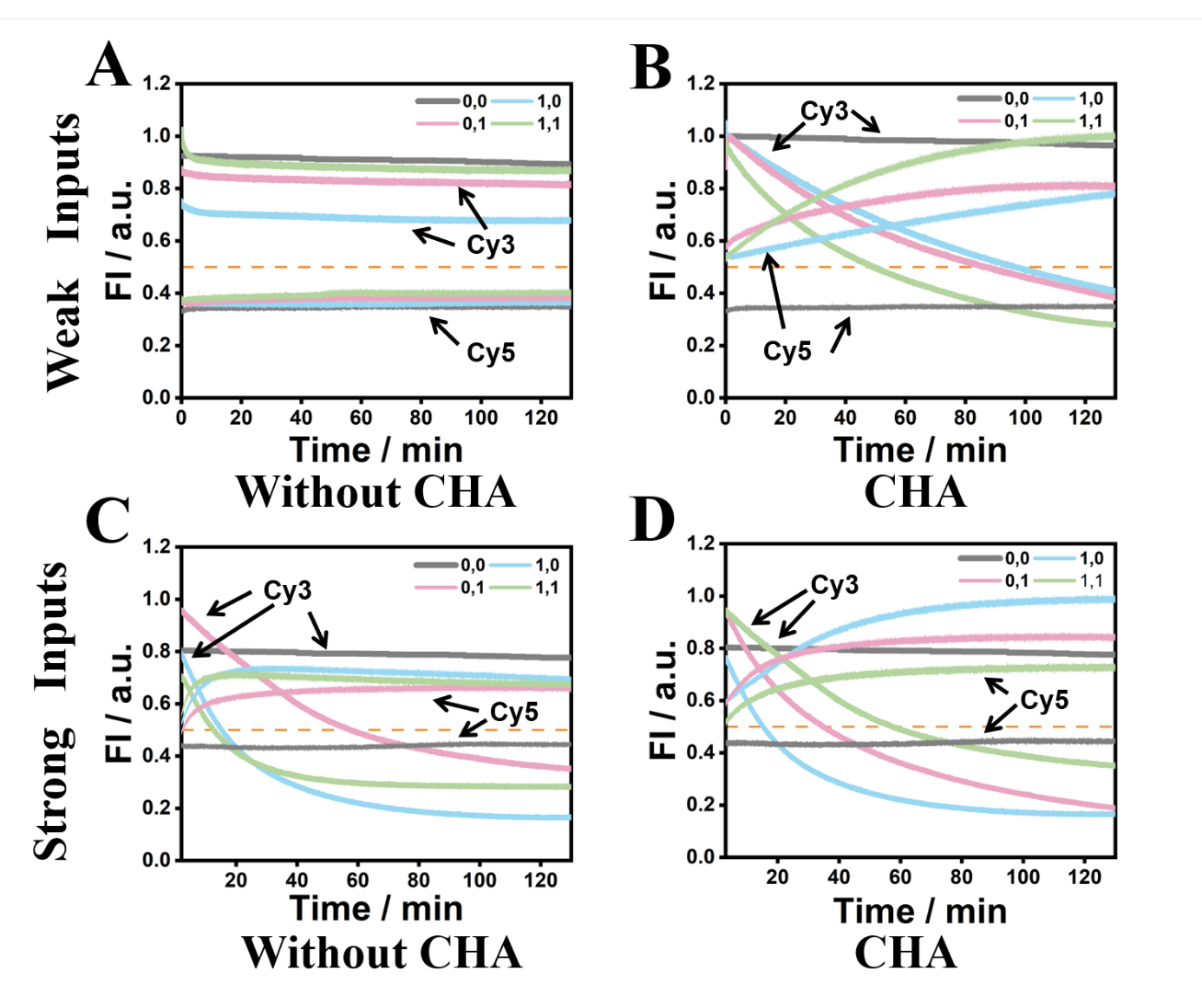


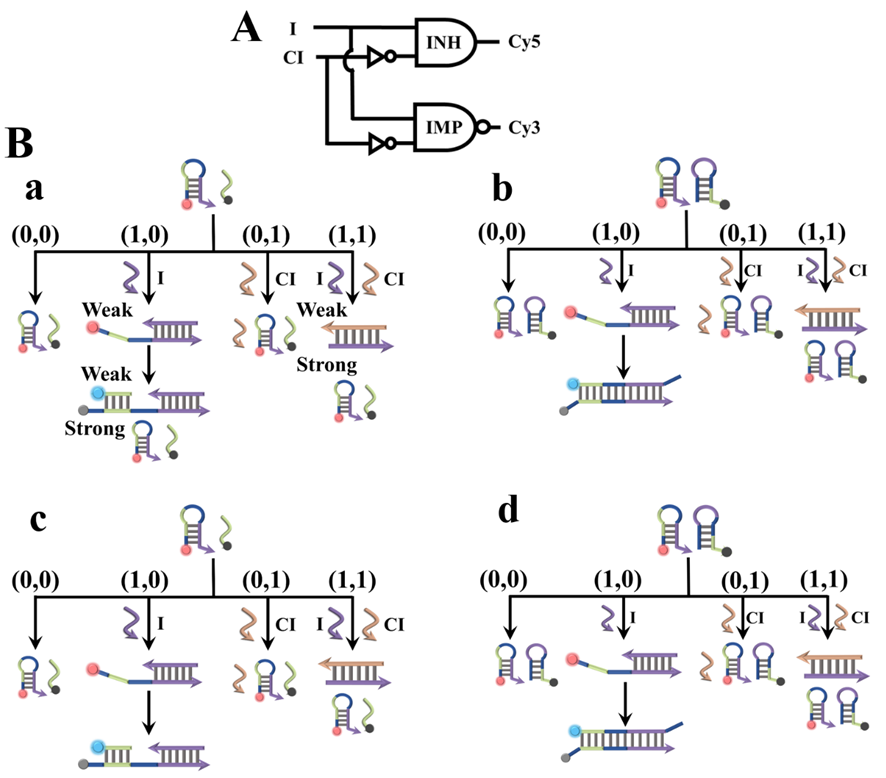
**Figure S9. (**A) Equivalent logic symbol of INH∧IMP CLP. **(**B) Detailed DNA reactions under different input combinations in four states. State a (weak-input & No CHA); State b (weak-input & CHA); State c (strong-input & No CHA); State d (strong-input & CHA).


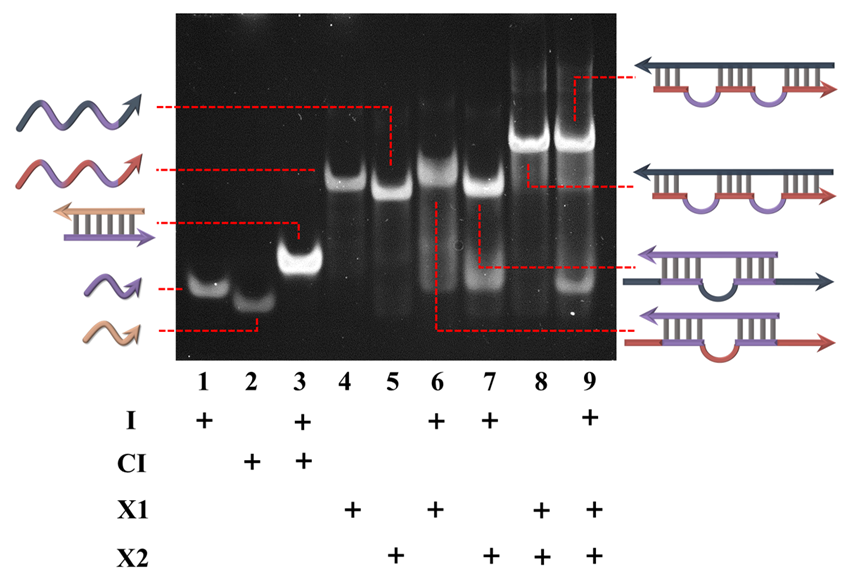
**Figure S10.** The PAGE results to confirm the assembly between different strands I, CI, X1 and X2.


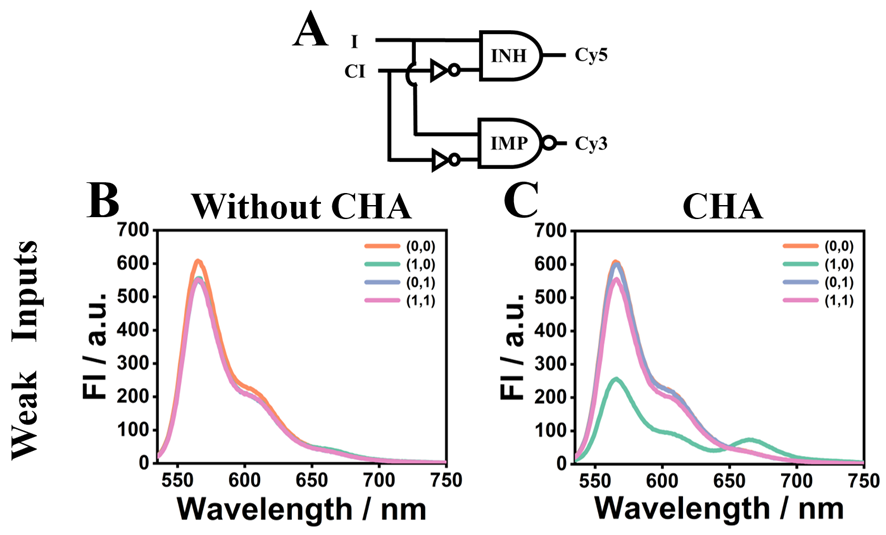
**Figure S11. (**A) Equivalent logic symbol of INH∧IMP CLP. (B) Fluorescence spectra in response to state a’. (C) Fluorescence spectra in response to state b’.


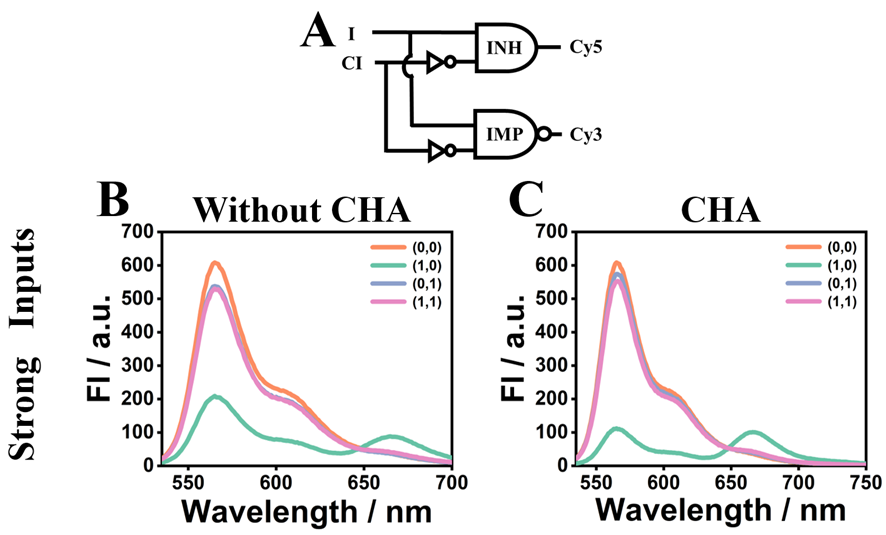
**Figure S12. (**A) Equivalent logic symbol of INH∧IMP CLP. (B) Fluorescence spectra in response to state c’. (C) Fluorescence spectra in response to state d’.


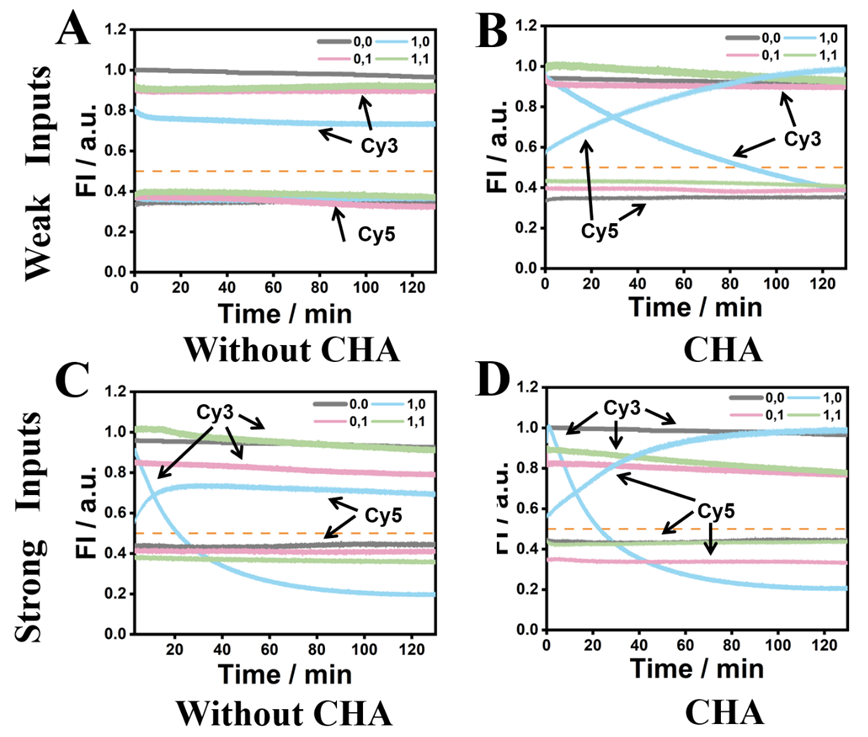
**Figure S13.** The kinetic fluorescence changes of Cy3 and Cy5 of the INH∧IMP CLP under 4 orthogonal states. **(**A) weak input & No CHA, (B) weak input &CHA, (C) strong input & No CHA, (D) strong input & CHA.


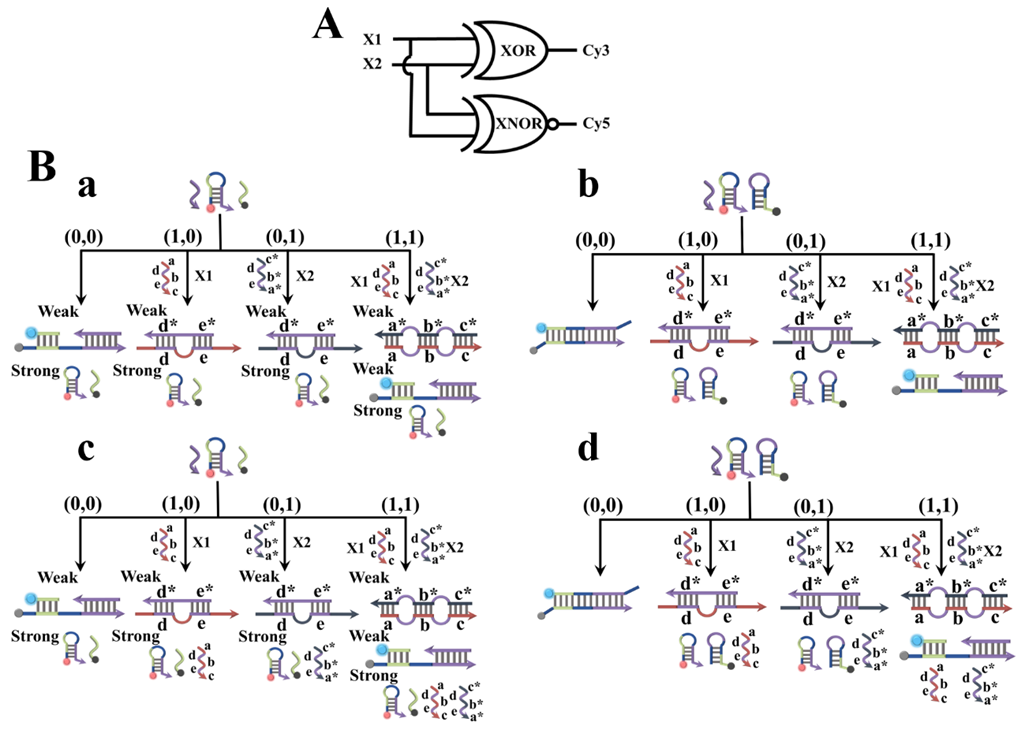


**Figure S14. (**A) Equivalent logic symbol of XOR∧XNOR CLP. **(**B) Detailed DNA reactions under different input combinations in four states. State a (weak-input & No CHA); State b (weak-input & CHA); State c (strong-input & No CHA); State d (strong-input & CHA).


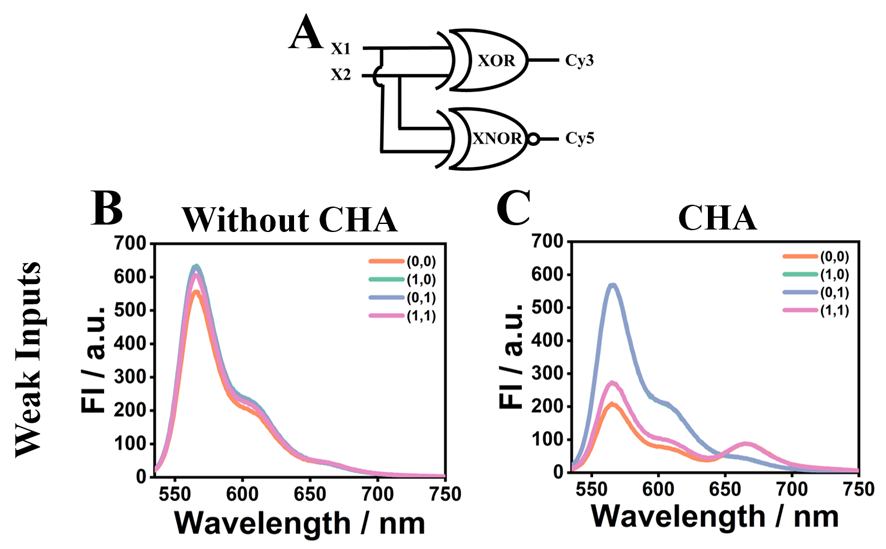
**Figure S15. (**A) Equivalent logic symbol of XOR∧XNOR CLP. (B) Fluorescence spectra in response to state a’. (C) Fluorescence spectra in response to state b’.


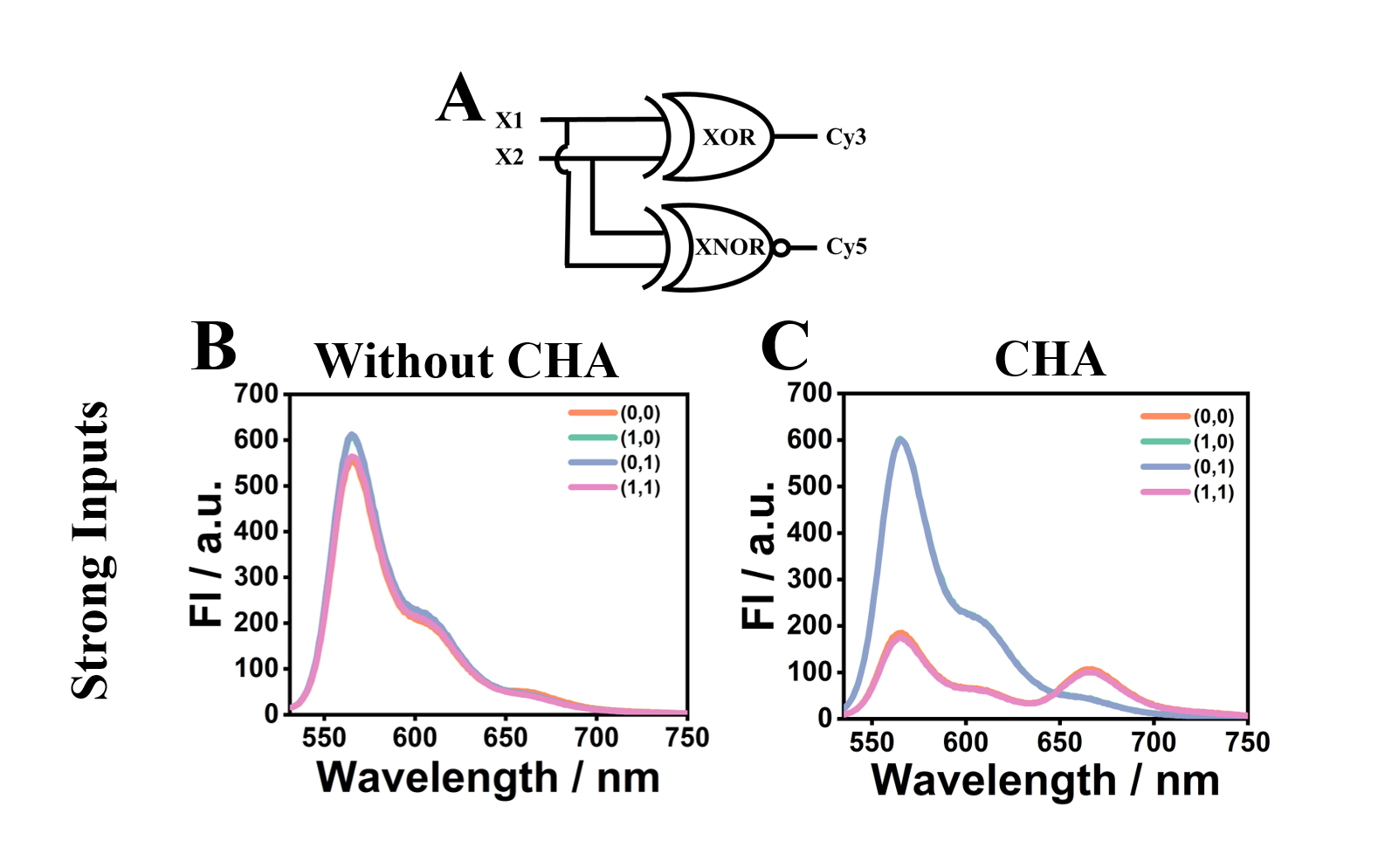
**Figure S16. (**A) Equivalent logic symbol of XOR∧XNOR CLP. (B) Fluorescence spectra in response to state c’. (C) Fluorescence spectra in response to state d’.


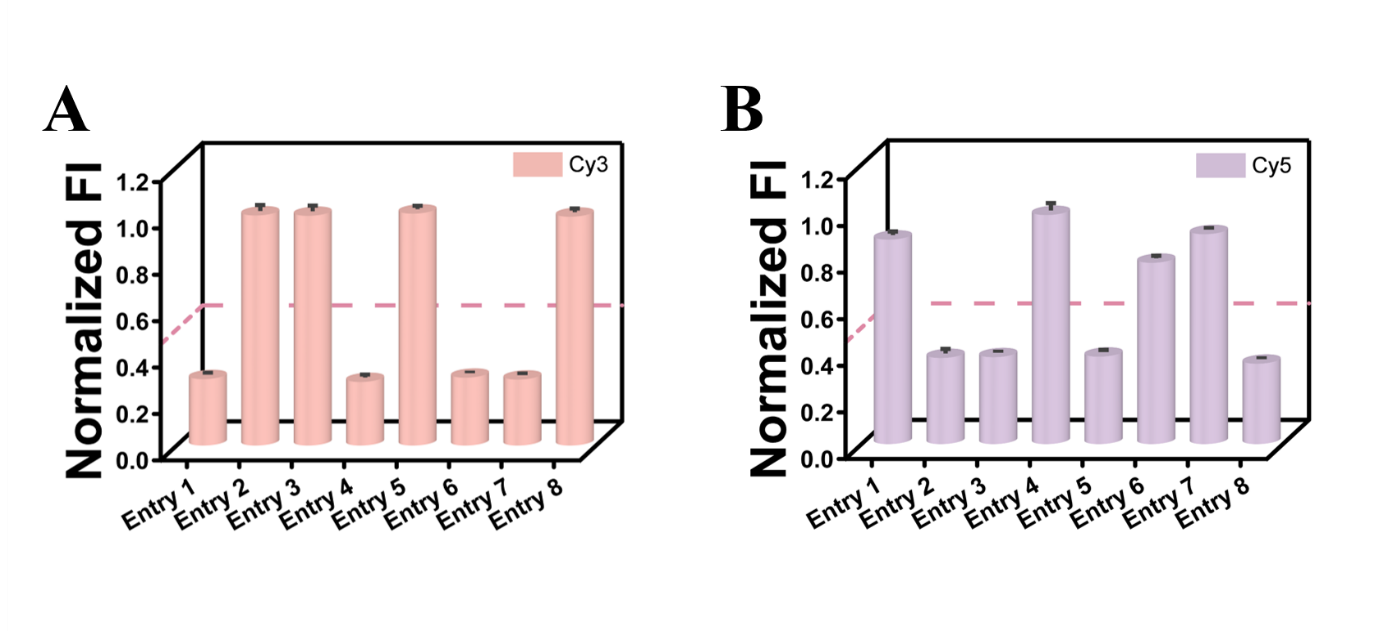


**Figure S17. (A)** Normalized fluorescence intensities of Cy3 at 567 nm for all the entries of even pC. (B) Normalized fluorescence intensities of Cy5 at 667 nm for all the entries of odd pC. The red dashed line represents the threshold value (0.5). All the error bars were obtained via three independent experiments.


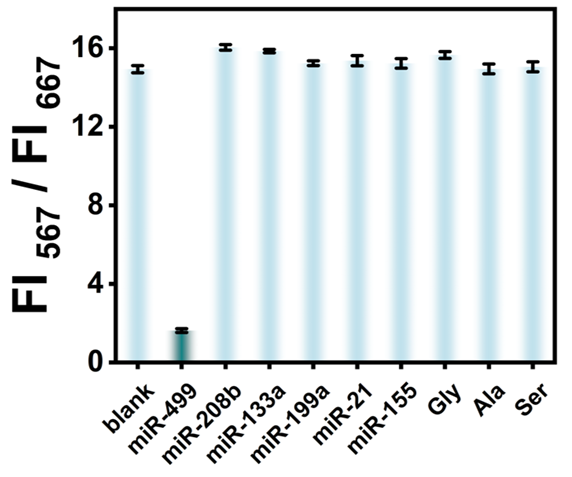


**Figure S18.** Selectivity of the system towards different miRNAs and several amino acids. The error bars were obtained via three independent experiments.


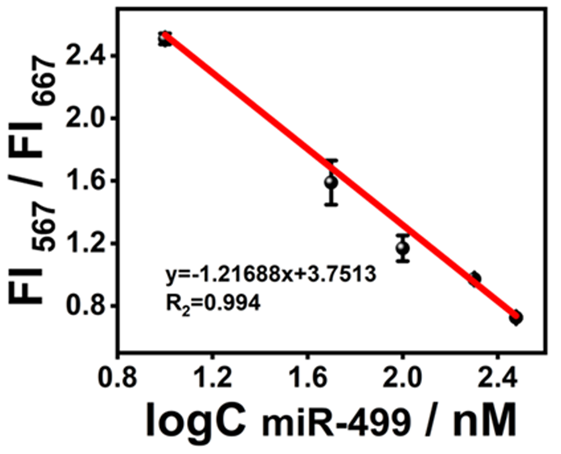


**Figure S19.** Linear relationship between the FI_567_/FI_667_ ratios and logarithmic values of the concentrations of miR-499, from 10 nM-300 nM. All the error bars were obtained via three independent experiments.


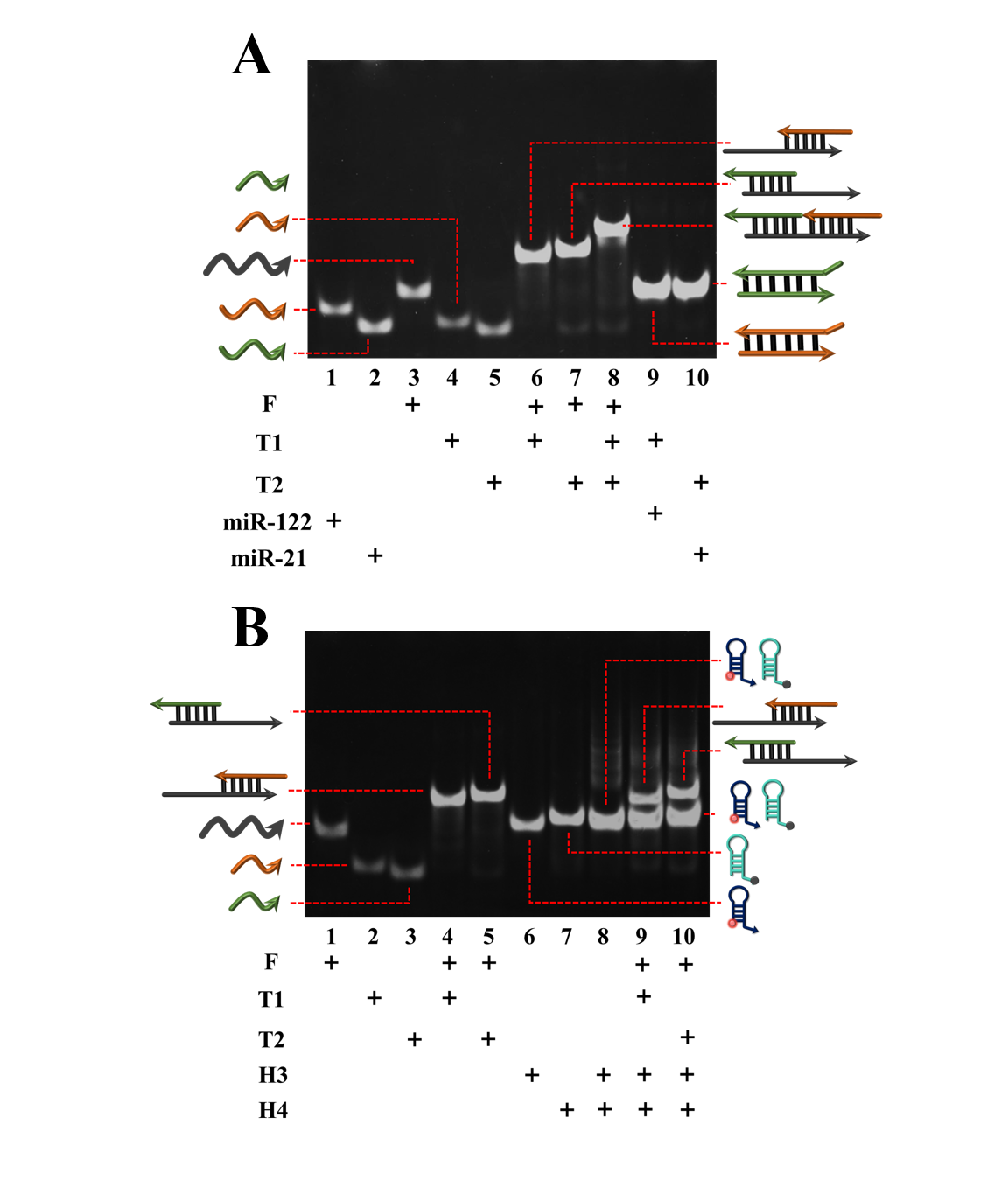


**Figure S20.** PAGE imaging results to identify the DNA reactions between (A) F, T1, T2, miR-122 and miR-21 and (B) F, T1, T2, H3 and H4, respectively. (The bands of H3 and H4 showed partial overlap due to similar structures)

**Table S2.** Comparison of different methods for the detection of typical miRNAs.

| Signal  amplification  strategy | Amplification cycles | Detection Methods | Target | LOD | Ref. |
| --- | --- | --- | --- | --- | --- |
| CHA | 1 | Fluorescent | miR-21 | 330 pM | 1 |
| CHA | 1 | Ratiometric fluorescent | miR-122 | 72 pM | 2 |
| CHA | 1 | Fluorescent | miR-141 | 297.1 pM | 3 |
| CHA | 1 | Fluorescent | miR-21 | 441.2 pM | 4 |
| CHA | 1 | Fluorescent | miR-122 | 510 pM | 5 |
| CHA-HCR | 2 | Chemiluminescence | miR-21 | 617 pM | 6 |
| Fe-MIL-88, CHA | 1 | Fluorescent | miR-499 | 25 pM | 7 |
| SDA, CRISPR-Cas12a | 2 | Fluorescent | miR-499 | 381.78 pM | 8 |
| CHA | 1 | Ratiometric fluorescent | miR-499 | 49 pM | This work |
|  |  |  | miR-122 | 99.8 pM |  |
|  |  |  | miR-21 | 86 pM |  |

**Table S3.** Recovery experiments for evaluating the reliability of detecting miR-499 with CHA in 1 ‰ human serum samples (mean ± SD, n = 3).

| Sample | Added(nM) | Measured(nM) | Recovery(%) | RSD(%) |
| --- | --- | --- | --- | --- |
| miR-499 | 3 | 2.89±0.075 | 96.3 | 2.6 |
|  | 7.5 | 7.25±0.064 | 96.6 | 0.9 |
|  | 75 | 73.55±3.091 | 98.1 | 4.2 |

**References**

[1] W. Ouyang, Z. Liu, G. Zhang, Z. Chen, L. Guo, Z. Lin, B. Qiu, G. Chen, *Anal. Methods* 2016, **8**, 8492.

[2] Y. Liu, T. Shen, J. Li, H. Gong, C. Chen, X. Chen, C. Cai, *ACS Sens.* 2017, **2**, 1430.

[3] H. Kima, S. Kanga, K. S. Park, H. G. Park, *Sens. Actuators, B* 2018, **260**, 140.

[4] J. Wu, Y. Tian, L. He, J. Zhang, Z. Huang, Z. Luo, Y. Duan, *Analyst* 2021, **146**, 3041.

[5] J. Yang, Y. Yu, Y. Cao, M. Guo, B. Lin, *Spectrochim. Acta, Part A* 2024, **314**, 124192.

[6] R. Wang, L. Xu, J. Li, *Sens. Actuators, B* 2024, **401**, 134965.

[7] X. Cheng, D. Ren, G. Xu, F. Wei, J. Yang, J. Xu, L. Wang, Q. Hu, Y. Cen, *Biosens. Bioelectron.* 2022, **196**, 113706.

[8] P. Li, Y. Ye, Y. Li, Z. Xie, L. Ye, J. Huang, *Biosens. Bioelectron.* 2024, **251**, 116129.
